# RelB contributes to the survival, migration and lymphomagenesis of B cells with constitutively active CD40 signaling

**DOI:** 10.3389/fimmu.2022.913275

**Published:** 2022-08-30

**Authors:** Laura B. Kuhn, Stefanie Valentin, Kristina Stojanovic, Daniel C. Strobl, Tea Babushku, Yan Wang, Ursula Rambold, Laura Scheffler, Sonja Grath, Dorothy John-Robbert, Helmut Blum, Annette Feuchtinger, Andreas Blutke, Falk Weih, Daisuke Kitamura, Roland Rad, Lothar J. Strobl, Ursula Zimber-Strobl

**Affiliations:** ^1^ Institute of Lung Health and Immunity, Helmholtz Zentrum München, German Research Center for Environmental Health, Neuherberg, Germany; ^2^ Institute of Computational Biology, Helmholtz Zentrum München, German Research Center for Environmental Health, Neuherberg, Germany; ^3^ Institute of Molecular Oncology and Functional Genomics, Technical University of Munich (TUM) School of Medicine, Technical University of Munich, Munich, Germany; ^4^ Institute of Asthma and Allergy Prevention, Helmholtz Zentrum München, German Research Center for Environmental Health., Munich, Germany; ^5^ Division of Evolutionary Biology, Faculty of Biology, Ludwig-Maximilians-Universität (LMU), Planegg-Martinsried, Germany; ^6^ Laboratory for Functional Genome Analysis, Gene-Center, Ludwig-Maximilians-Universität (LMU), Munich, Germany; ^7^ Research Unit Analytical Pathology, Helmholtz Zentrum München - German Research Center for Environmental Health, Neuherberg, Germany; ^8^ Research Group Immunology, Leibniz Institute on Aging - Fritz Lipmann Institute, Jena, Germany; ^9^ Research Institute for Biomedical Sciences, Tokyo University of Science, Noda, Japan; ^10^ TranslaTUM, Center for Translational Cancer Research, Technical University of Munich, Munich, Germany; ^11^ Cancer Consortium (DKTK), Heidelberg, Germany

**Keywords:** CD40, non-canonical NF-ĸB-signaling, RelB, IL9R, transgenic mice, B cell lymphoma, migration, LILRB4

## Abstract

Activation of CD40-signaling contributes to the initiation, progression and drug resistance of B cell lymphomas. We contributed to this knowledge by showing that constitutive CD40-signaling in B cells induces B cell hyperplasia and finally B cell lymphoma development in transgenic mice. CD40 activates, among others, the non-canonical NF-ĸB signaling, which is constitutively activated in several human B cell lymphomas and is therefore presumed to contribute to lymphopathogenesis. This prompted us to study the regulatory role of the non-canonical NF-ĸB transcription factor RelB in lymphomagenesis. To this end, we crossed mice expressing a constitutively active CD40 receptor in B cells with conditional RelB-KO mice. Ablation of RelB attenuated pre-malignant B cell expansion, and resulted in an impaired survival and activation of long-term CD40-stimulated B cells. Furthermore, we found that hyperactivation of non-canonical NF-кB signaling enhances the retention of B cells in the follicles of secondary lymphoid organs. RNA-Seq-analysis revealed that several genes involved in B-cell migration, survival, proliferation and cytokine signaling govern the transcriptional differences modulated by the ablation of RelB in long-term CD40-stimulated B cells. Inactivation of RelB did not abrogate lymphoma development. However, lymphomas occurred with a lower incidence and had a longer latency period. In summary, our data suggest that RelB, although it is not strictly required for malignant transformation, accelerates the lymphomagenesis of long-term CD40-stimulated B cells by regulating genes involved in migration, survival and cytokine signaling.

## Introduction

In mammals, the Nuclear Factor (NF)-κB family consists of five proteins: RelA, c-Rel and RelB, which have a transactivation domain, and complex with p50 or p52 to form transcriptionally active heterodimers in the nucleus. In resting B cells, NF-ĸB proteins are retained in the cytoplasm, and thereby remain transcriptionally inactive. Two NF-ĸB pathways have been identified, designated as canonical and non-canonical. Receptors like the B-cell receptor (BCR), CD40 or Toll-like receptors (TLRs) induce canonical NF-κB signaling by activating the inhibitor kinase complex Ikkγ/Ikkα/Ikkβ, resulting in phosphorylation and degradation of Iκb-α and thereby the release and translocation of RelA/p50 or c-Rel/p50 heterodimers into the nucleus. Members of the Tumor Necrosis Factor Receptor (TNF-R) family, e.g. CD40 or BAFF-R, additionally activate the non-canonical NF-κB pathway by stabilizing the NF-ĸB kinase (NIK), which in turn phosphorylates Ikkα, which subsequently phosphorylates the cytoplasmic p100 (NF-ĸB2). Phosphorylated p100 is cleaved to p52, which is then translocated as p52/RelB heterodimer into the nucleus (1–3). RelB plays an essential role in the immune system. Disruption of the RelB locus in all cells (complete RelB-KO) results in multiorgan inflammation, disruption of splenic architecture, and impaired germinal center formation (4, 5). Some years ago, the B cell intrinsic function of RelB and p52 has been studied by analyzing mice with conditional alleles of RelB and NF-ĸB2. A marked reduction of B cells was observed in RelB/NF-ĸB2 double-deficient mice, whereas single deletion of RelB led to only a slight decrease of mature B cells (6). Furthermore, a germinal center specific double deletion of RelB/NF-ĸB2 resulted in the collapse of germinal center B cells and a defect in plasma cell differentiation (7). A similar phenotype was described in mice with B cell specific inactivation of NIK (8). In the same year, Almaden and colleagues published a study describing the phenotype of RelB/c-Rel double deficient mice by generating mixed bone marrow chimeras. They observed decreased B cell numbers in the periphery after transplantation of double deficient but not of single deficient hematopoietic stem cells. They concluded that BAFF-R stimulation activates both RelB and c-Rel containing dimers, which coordinately ensure the development and survival of peripheral B cells (9). Since single inactivation of RelB in mature resting B cells had merely a minor effect on B cell biology in these studies, the authors concentrated on describing the phenotype of double deficient B cells.

Activation of non-canonical NF-ĸB signaling must be well balanced to guarantee B cell survival without inducing chronic stimulation, which might induce B cell lymphomas. Thus, constitutive activation of non-canonical NF-ĸB signaling was observed in various B cell lymphomas such as Chronic Lymphocytic Leukemia (B-CLL), Mantle Cell Lymphoma (MCL), Diffuse Large B Cell Lymphoma (DLBCL) and Hodgkin-Lymphomas (HL) (10–16). Recently, a new subset of DLBCL has been described, characterized by RelB activation (17). Hyperactivation of non-canonical NF-κB signaling can be caused by genetic mutations in key signaling molecules (e.g. BIRC3, NIK, TRAF2 and TRAF3) (12) or through stimulation by cells of the microenvironment (18). Mouse models mimicking genetic alterations in the non-canonical NF-κB pathway, such as inactivating mutations of TRAF2, TRAF3 and CIAP, or overexpression of NIK, exhibit a B-cell accumulation and/or B-cell lymphoma development (19–22). Although these findings hint at an important role of the non-canonical NF-ĸB signaling in the development of B cell lymphomas the contribution of RelB to lymphopathogenesis is still largely unknown.

The starting point for B cell lymphomas driven by non-canonical NF-ĸB signaling may be chronic microbial or viral infections as well as autoimmune diseases, in which activated T cells, expressing the CD40-L, are recruited into the inflammatory environment. This can lead to the continuous CD40-stimulation of B cells, resulting in their improved survival and proliferation (23). These chronically stimulated B cells are at risk of becoming malignant by acquiring secondary mutations (24). Moreover, continuous CD40-stimulation of lymphoma cells contributes to lymphoma cell survival, proliferation and drug resistance (25, 26). These data suggest that deregulated CD40 signaling is involved in lymphoma initiation and progression. To investigate the contribution of RelB in the process of lymphomagenesis, we genetically disrupted RelB in LMP1/CD40 mice. These mice express a fusion protein consisting of the transmembrane domain of the Epstein-Barr viral latent protein 1 (LMP1) and the intracellular signaling part of CD40 in B cells. LMP1/CD40-expression leads to constitutive activation of CD40 and mainly activation of the non-canonical NF-ĸB pathway and to a lesser extent of canonical NF-κB signaling. This results in a premalignant B cell expansion in young mice and in B cell lymphoma development in aged mice (27). Since this mouse model substantially mimics the constitutive CD40-stimulation of B cells in a pathological situation in humans, we regarded it as a perfect tool to study the role of RelB and its regulated genes in the initiation and progression of B cell lymphomas.

## Materials and methods

### Mice

RelB^fl/fl^, LMP1/CD40^stopfl^, CD19-Cre mice have been recently described (27–29). For generation of mice with B cell specific deletion of RelB, we mated RelB^fl/fl^ mice with CD19-Cre^+/-^ mice resulting in genetic disruption of RelB in B cells from the early B cell development onwards. In combination with the CD19-Cre strain, the deletion efficiency of floxed alleles is rather low in B cells from the BM (~ 40%) but increases in peripheral B cells to ~95%. In addition, we generated mice expressing LMP1/CD40 in B cells in the presence and absence of RelB. The following mice were analyzed: LMP1/CD40^stopfl^//CD19-Cre^+/-^ (LMP1/CD40), LMP1/CD40^stopfl^//RelB^fl/fl^//CD19-Cre^+/-^ (LMP1/CD40//RelB-KO), and CD19-Cre^+/^. All mouse strains were on a Balb/C background. Control and mutant mice were always examined in parallel. Mice were analyzed at an age between 10 and 16 weeks if not otherwise stated. Primers used in genotyping are listed in **Supplementary Table 1**. Mice were maintained in specific pathogen-free conditions. Experiments were performed in accordance with the German Animal Welfare Act and were approved by the institutional committee on animal experimentation and the Government of Upper-Bavaria.

### Flow cytometry (FACS)

Antibodies used for FACS are listed in **Supplementary Table 2**. For the detection of IL-9, MACS-purified CD4^+^ T cells were incubated with 1x protein transport inhibitor cocktail (Invitrogen, Thermo Fisher Scientific), Ionomycin (500ng/ml, LIFE Technologies) and Phorbol 12-myristate 13-acetate (PMA) (50 ng/ml, Sigma Aldrich) for 4h at 37°C. Intracellular (IC) FACS staining was performed using the Fixation/Permeabilization Solution Kit (Invitrogen, Thermo Fisher Scientific), according to manufacturer´s manual. To exclude dead cells, the LIVE/DEAD fixable dead cell stain (Invitrogen, Thermo Fisher Scientific) or a TO-PRO-3 (Invitrogen, Thermo Fisher Scientific) staining was performed. To discriminate viable cells and early apoptotic cells from late apoptotic and dead cells, an Annexin V and 7AAD staining in combination with a CD21/CD23 staining was performed. For the Annexin V/7-AAD staining the PE-Annexin V Apoptosis detection kit (BD Pharmingen) was used. Following cell surface antibody staining for 25 min on ice, 1x10^6^ cells were taken in 100 µL 1x Annexin V binding buffer and stained with 5 µL Annexin V – PE and 5 µL 7AAD for 15 min at RT in the dark, according to the manufacturer´s protocol. Cell samples were acquired within 30 min. FACS data were acquired with a FACSCalibur™ or a LSRII Fortessa Flow Cytometer (Becton Dickinson). Results were evaluated using FlowJo (TreeStar).

### Stainings of paraffin-sections and immunohistochemistry

Stainings were performed as described in (30). Antibodies are listed in **Supplementary Table 3**.

### Cell purification and *in vitro* cultures

B and T cells were isolated by magnetic cell sorting (Miltenyi Biotec, Bergisch-Gladbach, Germany): Splenic B cells were isolated with CD43^+^ beads, B cells for the 7AAD/Annexin V staining and lymphoma cells with the Pan B cell isolation Kit to avoid the loss of CD43^+^ B cells. CD4^+^ T cells were isolated with the murine CD4^+^ T Cell Isolation Kit. For proliferation and survival assays, splenic B cells were cultivated for up to five days in RPMI medium containing 10% (v/v) heat inactivated fetal calf serum (FCS) (PAA Cell culture Company), 1% (v/v) penicillin/streptomycin, 1% (v/v) sodium pyruvate, 1% (v/v) L-glutamine, 50 µM β-mercaptoethanol. Except for FCS, all supplements were purchased from Gibco. The following agents were used for stimulation of B cells: α-CD40 antibody (2.5 μg/ml, eBioscience [HM40-3]), recombinant IL-9 (10 ng/mL, eBioscience), IL-4 (10 ng/ml, Sigma-Aldrich) and BAFF (100 ng/ml, R&D). For proliferation assays, B cells were stained with 5 µM 5-(and 6)-carboxyfluorescein diacetate N-succinimidyl ester (CFSE) (Molecular Probes) for five minutes at 37°C before culturing them. For analysis of apoptosis, B cells were cultured up to three days in the absence of stimulation. At each day (d1-3) an 7AAD/Annexin V staining in combination with a CD21 and CD23 staining was performed. To be able to better study apoptosis in the aberrant CD21^−^CD23^−^ population, we prepared B cells from old mice (between 7 and 10 months).

### Western blot analysis

Protein extract preparation and Western blot analysis were performed as previously described (27, 31). Antibodies are listed in **Supplementary Table 3**. For the nuclear fractionations, 2x10^7^ B cells were incubated for 15 min in 100 µl buffer A (10 mM HEPES pH 7.9: 10 mM KCL; 0.1 mM EDTA; 0.1 mM EGTA; 1 mM DTT; 1x Protease inhibitor (Mini complete, Roche) on ice. Subsequently, 6.25 μl 10% NP-40 (Sigma-Aldrich) was added to each sample and the samples were shaken for 5 min at 4°C on a vortexer. Afterwards, the nuclei were spun down (10.000xg) for 15 min. The supernatant contained the cytoplasmic fraction and was stored at -80°C. The nuclei were washed in 1.5 ml Buffer A. After centrifugation, the pellet was mixed with 40 μl of buffer C (20 mM HEPES pH 7.9; 0.4 M NaCL; 1 mM EDTA; 1 mM EGTA; 1 mM DTT; 1x protease inhibitor (Mini complete, Roche) to lyse the nuclei. Samples were then shaken on a vortexer for 30 min at 4°C. After centrifugation (10.000xg for 15 min) the supernatant, which contained the nuclear fraction, was stored at -80°C.

### Quantification of the MZ area

The IgM^+^ B cells, located in the MZ outside the follicle, delineated by a Moma1^+^ macrophage ring, were quantified using ImageJ (1.50e). The MZ area was compared between LMP1/CD40 and LMP1/CD40//RelB-KO animals. In a first step, re-calibration of the 100 µM scale bar on previously acquired images from 5 splenic sections was performed using the straight line tool. Next, the inside area of the B cell follicle along the macrophage ring (a), and the area of the full B cell follicle including the outer MZ (b) were measured using the polygon selection tool. Subtraction of the inner follicular area (a) from the total B cell follicle area (b) generated values for the area of the outer MZ (c=b-a). Lastly, the percentage of the MZ in each follicle was calculated by building the ratio between the outer MZ area (c) and total B cell follicle area (b) * 100. The MZ percentages were quantified from 7 follicles per genotype and were plotted and analyzed for statistical significance in GraphPad Prism (v8).

### RNA-Seq analysis

For RNA preparation, B lymphocytes were isolated from the spleens of 8-11-week old male mice by MACS purification using CD43^+^ magnetic beads (Miltenyi Biotec, Bergisch-Gladbach, Germany). RNA-Seq was performed with eight independent replicas for LMP1/CD40 and four independent replicas each of LMP1/CD40//RelB-KO and CD19-Cre. One LMP1/CD40//RelB-KO sample was excluded from further analysis due to technical issues in cell preparation. For the isolation of total RNA, 1 x 10^7^ B cells (>95% CD19-positive cells) were lysed in TRIzol Reagent (Thermo Fisher Scientific Inc.). RNA was isolated using Direct-zol™ RNA MiniPrep kit (Zymo Research) according to manufacturer’s instructions. RNA quality was analyzed by Bioanalyzer microcapillary electrophoresis on RNA Nano chips. RNA integrity numbers were >9. Sequencing libraries were constructed with the Encore™ Complete RNA-Seq DR Library System kit (NuGen) to capture all non-ribosomal RNA. Single-end 100 bp sequencing was performed by a HiSeq1500 (Illumina) to an average sequencing depth of 30 x 10^6^ reads per sample for the comparison LMP1/CD40 vs LMP1/CD40//RelB-KO. Paired end sequencing (2 x 60 b) was done for the CD19-Cre vs LMP1/CD40 comparison at the same sequencing depth on a NextSeq 1000 (Illumina). The raw read data were first filtered with rrnafilter (32) to remove ribosomal RNA and then mapped to the reference (GRCm38.89) with hisat2 (33). To generate the gene expression matrix and count the reads, featureCounts was used (34). These three steps were managed and run *via* Watchdog (35). We calculated differential gene expression between the condition with DESeq2 (36). The code of the analysis is provided on GitHub [https://github.com/danielStrobl/relB_analysis]. The heatmap was generated with Clustvis (37). All Gene Set Enrichment Analyses (GSEA) were performed with GSEA v4.0.2 from Broad Institute (38, 39). The Gene Ontology Overrepresentation Test was performed with the online tool at [http://pantherdb.org/] (40). Correlation analysis of the differentially expressed genes between LMP1/CD40 vs LMP1/CD40-RelB-KO and CD19-Cre vs LMP1/CD40 was done by the simple linear regression tool from Graphpad Prism 9.4. Differentially expressed genes from the LMP1/CD40 vs CD19-Cre comparison were filtered by a threshold of p_adjust_ < 0.05.

### RT-Pcr

RNA was prepared with the RNeasy Mini Kit (Qiagen). To obtain cDNA, the QuantiTec Reverse Transcription Kit (Quiagen) was used. RT-qPCR was performed using the LightCycler 480 Probes Master Mix (Roche) according to the manufacturer´s protocol and a LightCycler 480 II (Roche Diagnostics). Primers and probes were designed with the Universal Probe Library Assay Design Center provided by Roche Diagnostics. RNAPol2 and YWHAZ were used as reference genes. Complete primer sequences and probes are presented in **Supplementary Table 1**.

### Southern blot

To detect monoclonal B cell expansions, Southern blots were performed with a radioactive probe spanning the J_H1-4_ region of the IgH locus as previously described (27).

### Statistics

The Prism software (versions 7-9, GraphPad software) was used to perform all the statistical analyses. Specific statistical analysis methods are indicated in the figure legends.

## Results

### B cell specific inactivation of RelB has a minor effect on peripheral B cell numbers

In order to explore the B cell-intrinsic functions of RelB in B cell development and maintenance, we analyzed RelB^fl/fl^//CD19-Cre mice (hereafter referred to as RelB-KO mice) (28). We confirmed the deletion of RelB in MACS-purified splenic B lymphocytes by Western blot analysis (**Supplementary Figure 1A**). In addition, the Western blot analysis revealed reduced amounts of p100 and p52 in the B cells from RelB-KO, in comparison to control mice (**Supplementary Figure 1B**).

**Figure 1:**
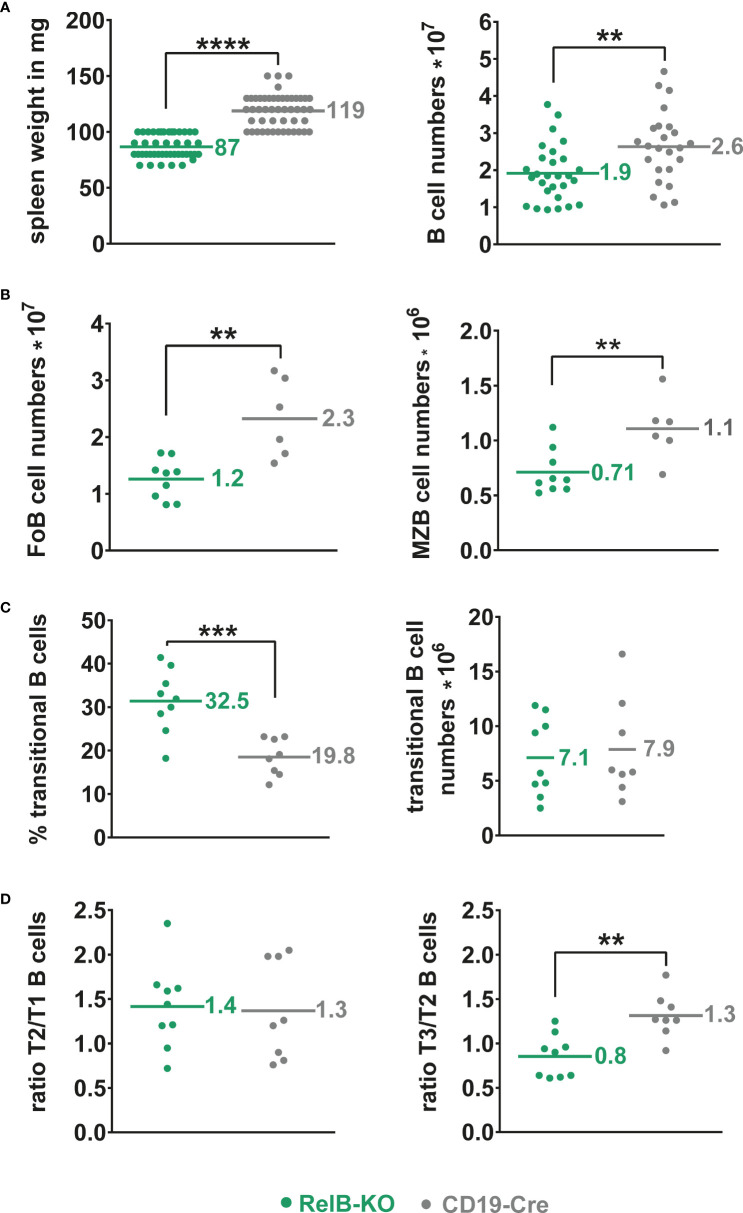
Slightly reduced B cell numbers in the spleen of RelB-KO mice: **(A)** Splenic weight and splenic B cell numbers (CD19^+^) of RelB-KO and CD19-Cre mice (controls). *N*≥ 24 mice. **(B)** Total numbers of MZB (CD21^high^CD23^low^) and FoB (CD21^int^CD23^high^) cells gated as indicated in Suppl. Figure 3B. *N*≥ 6 mice. (**A, B**) Unpaired two-tailed t-test was performed. Due to their log-normal distribution the values were logarithmized before statistical analyses. **(C)** Percentages and total numbers of B220^+^AA4.1^+^ transitional B cells *N*≥ 8 mice. Statistical analyses were conducted with an unpaired two-tailed t-test with Welch´s correction for the percentages, and with an unpaired two-tailed t-test after logarithmic transformation for cell numbers. **(D)** Ratio of the percentages of T2 to T1 and T3 to T2 cells in each mouse. *N*≥ 8 mice. Unpaired two-tailed t-test was performed. Dots represent values from individual mice, and the lines and numbers indicate the means. ** P<0.01, *** P<0.001, **** P<0.0001.

Unchanged B cell numbers and percentages of developing B cells in the bone marrow (BM) suggested a normal B cell development in RelB-KO mice. However, mature recirculating B cells were reduced to approximately half in RelB-KO relative to control mice (**Supplementary Figure 2**). In the periphery, the splenic weight and total B cell numbers were slightly but significantly reduced in RelB-KO, in comparison to control mice (**Figure 1A**), resulting in proportionally more T than B cells in comparison to control mice (**Supplementary Figure 3A**). The B cell reduction affected both Follicular B (FoB) cells and Marginal zone B (MZB) cells (**Figure 1B**, **Supplementary Figure 3B**). The percentages of transitional AA4.1^+^ B cells were increased in RelB-KO mice resulting in comparable numbers of transitional B cells like in control mice (**Figure 1C**). Relative to controls, the percentages of T3 B cells were decreased in RelB-KO mice (**Supplementary Figure 3C**), resulting in decreased T3/T2 and similar T2/T1 ratios in comparison to controls (**Figure 1D**). These data suggested an impaired differentiation of T2 cells into T3 and mature B cells. In the blood, percentages of B and T lymphocytes were comparable between RelB-KO mice and controls (**Supplementary Figure 3D**). However, the percentage of transitional B cells was once again higher in RelB-KO compared to control mice (**Supplementary Figure 3E**). In addition, percentages of B cells in inguinal lymph nodes (**Supplementary Figure 3F**) and B1a cells in the peritoneal cavity were reduced in RelB-KO relative to control mice (**Supplementary Figure 3G**). These data suggest that inactivation of RelB results in a slight but significant decrease of mature B cells, both from the B2 and B1 cell subtype.

### B cells are yet expanded and activated in the spleen of LMP1/CD40 mice despite the inactivation of RelB

**Figure 2:**
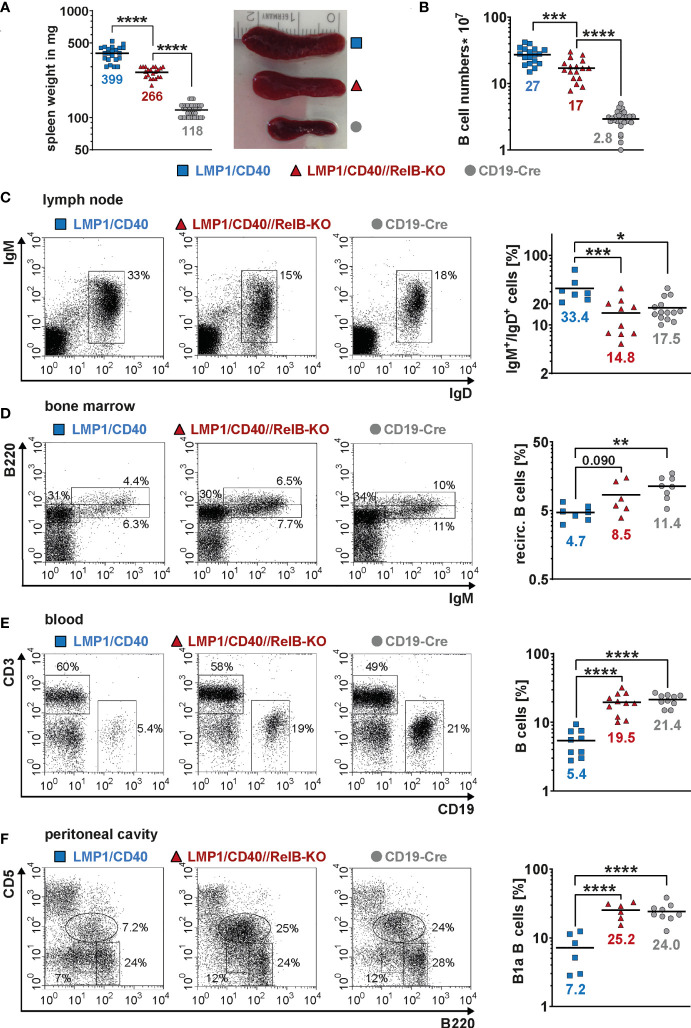
Inactivation of RelB in LMP1/CD40 mice leads to reduced B cell numbers in secondary lymphoid organs: **(A)** Splenic weight and size of the indicated genotypes. *N*≥21. **(B)** Splenic B cell numbers in the indicated genotypes. *N*≥17. **(C)** Percentages of IgM^+^IgD^+^ B cells in inguinal lymphnodes. FACS plots are pre-gated on live lymphocytes. LMP1/CD40 *N*=7; LMP1/CD40//RelB-KO *N*=10; CD19-Cre *N*=14. **(D)** Percentages of recirculating B cells in the BM. FACS plots are pre-gated on live lymphocytes. Recirculating B cells were determined as IgM^+^B220^high^. LMP1/CD40 *N*=7; LMP1/CD40//RelB-KO *N*=6; CD19-Cre *N*=8. **(E)** Percentages of B cells (CD19^+^) and T cells (CD3^+^) in the blood. Plots are pre-gated on live lymphocytes. The graph compiles the percentages of CD19^+^ B cells from different independent experiments. LMP1/CD40 *N*=9; LMP1/CD40//RelB-KO *N*=11; CD19-Cre *N*=10. **(F)** Percentages of B1a (B220^low^CD5^+^) and B2 (B220^+^CD5^−^) cells in the peritoneal lavage. FACS plots are pre-gated on live lymphocytes. The graph compiles the percentages of B1a cells. LMP1/CD40 *N*=6; LMP1/CD40//RelB-KO *N*=6; CD19-Cre *N*=9. **(A–F)** Due to their lognormal distribution values were logarithmized before statistical analysis with an ordinary one-way ANOVA, Tukey´s multiple comparisons test with * P<0.05, ** P<0.01, *** P<0.001, **** P<0.0001. **(A–F)** Symbols represent values from individual mice, and the lines and numbers indicate the means.

Next, we asked, whether inactivation of RelB affects the B cell expansion of LMP1/CD40^stopfl/wt^//CD19-Cre^+/−^ (LMP1/CD40) mice expressing a constitutive active CD40 receptor in B cells (27). Constitutive activity of CD40 was achieved by fusion of the C-terminal signaling part of CD40 to the transmembrane domain of the Epstein-Barr viral protein LMP1 that confers constitutive active ligand-independent activity of CD40 (41) (**Supplementary Figure 4A**). To inactivate RelB in B cells from LMP1/CD40 mice, we combined them with RelB^fl/fl^ mice (28) resulting in LMP1/CD40^stopfl/wt^//RelB^fl/fl^//CD19-Cre^+/−^ (LMP1/CD40//RelB-KO) mice (**Supplementary Figure 4B, C**). Western blot analysis confirmed strongly reduced levels of RelB in B cells from LMP1/CD40//RelB-KO mice (**Supplementary Figure 5** and **Supplementary Figure 6**). Furthermore, we analyzed the impact of RelB inactivation on the activity of the canonical and non-canonical NF-ĸB pathway. Inactivation of RelB led to a reduction in p100 levels (**Supplementary Figure 5A**). Nearly complete processing of the remaining p100 amounts resulted in basal p52 levels comparable to those of control mice (**Supplementary Figure 5A** and **Supplementary Figure 6**). Inactivation of RelB had no further major influence on Iĸ-B and pIĸB levels in LMP1/CD40-expressing cells, which were decreased in both LMP1/CD40 and LMP1/CD40//RelB-KO B cells in comparison to controls (**Supplementary Figure 5B**). Similar amounts of p50 and c-Rel and slightly increased levels of p65 in the nuclei of LMP1/CD40 compared with LMP1/CD40//RelB-KO mice suggest that the canonical NF-ĸB pathway is not further activated in order to compensate the loss of RelB (**Supplementary Figure 6**). In LMP1/CD40//RelB-KO mice, the size of the spleen, splenic weight and B cell numbers were still increased compared to control mice. However, numbers of examined parameters were reduced by roughly one third compared to RelB-proficient LMP1/CD40 mice (**Figures 2A, B**). In the lymph nodes, percentages of B cells were comparable between LMP1/CD40//RelB-KO and control mice, and thus reduced in comparison to LMP1/CD40 mice (**Figure 2C**). Instead, percentages of recirculating B cells in the BM and B cells in the blood were higher in LMP1/CD40//RelB-KO than in LMP1/CD40 mice, the latter showing a strong reduction of B cells compared to controls in both organs (**Figures 2D, E**). The peritoneal cavity of LMP1/CD40 mice harbored a lower percentage of B1a cells in comparison to controls, which was reverted by the inactivation of RelB (**Figure 2F**). Thus, we conclude that continuous activation of the non-canonical NF-κB pathway retains LMP1/CD40-expressing B cells in the B cell follicles of secondary lymphoid organs (SLO) and inhibits their migration into the blood and peritoneal cavity as well as their recirculation into the BM.

**Figure 3:**
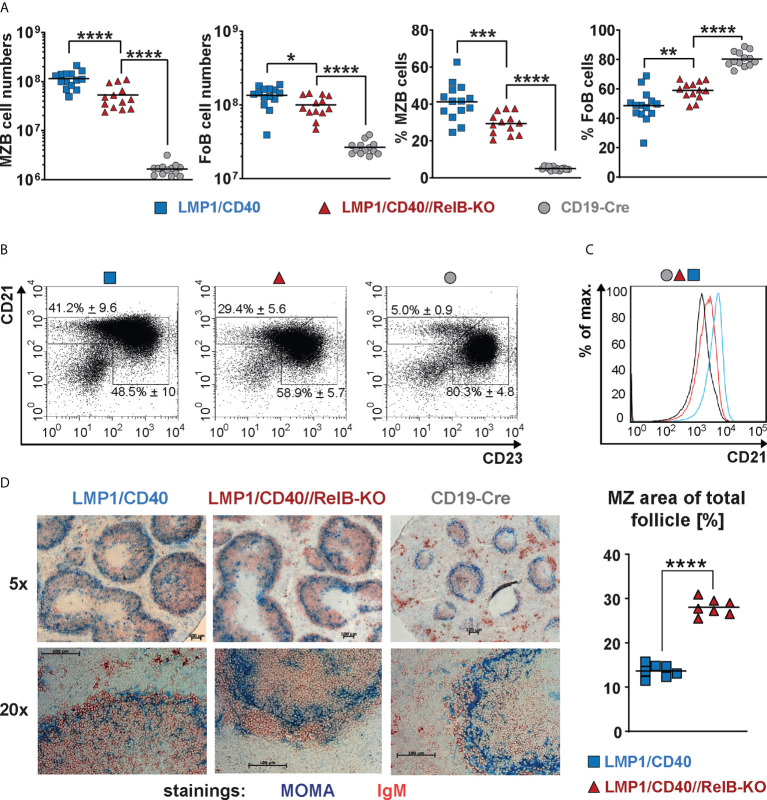
CD21^high^CD23^low^ cells are still expanded in RelB-deficient LMP1/CD40 mice: **(A)** Total cell numbers and percentages of FoB and MZB cells in the spleen. *N*≥13 individual mice per genotype. Ordinary one-way ANOVA with Tukey’s multiple comparison was performed with * P<0.05, ** P<0.01, *** P<0.001, **** P<0.0001. Values were logarithmized before statistical analysis. Symbols represent values from individual mice, and the lines indicate the means. **(B)** Representative FACS plots to differentiate MZB (CD21^high^CD23^low^) and FoB (CD21^+^CD23^+^) cells. FACS plots are pre-gated on live B220^+^ lymphocytes. **(C)** Overlay of the CD21 surface expression of splenic B lymphocytes from mice with the indicated genotypes. **(D)** Splenic sections from the indicated genotypes were stained with an anti-IgM (red) antibody to detect B cells and an anti-Moma-1 (blue) antibody to visualize metallophilic macrophages lining the Marginal Zone sinus. One representative experiment is shown; *N*=3 independent experiments. The graph depicts the percentages of the marginal zone area within the total follicle area from the indicated genotypes. Areas were determined in 7 different follicles using ImageJ as described in Materials and Methods. Supplementary Figure 7 shows an example of MZ zone delineation. Unpaired two-tailed t test was performed with **** P<0.0001.

Ablation of RelB diminished, but did not abolish the expansion of CD21^high^CD23^low^ MZB cells in LMP1/CD40 mice, although their total CD21 surface expression was decreased in comparison to LMP1/CD40 B cells (**Figures 3A–C**). Although the percentages of MZB cells were lower in LMP1/CD40//RelB-KO mice in comparison to LMP1/CD40 mice more B cells were located in the MZ (**Figure 3D**, **Supplementary Figure 7**). These data suggest that constitutive activation of the non-canonical NF-κB pathway preferentially supports the expansion of MZB cells but inhibits their migration into the MZ.

### Inactivation of RelB impairs the survival and activation of CD40-stimulated B cells

**Figure 4:**
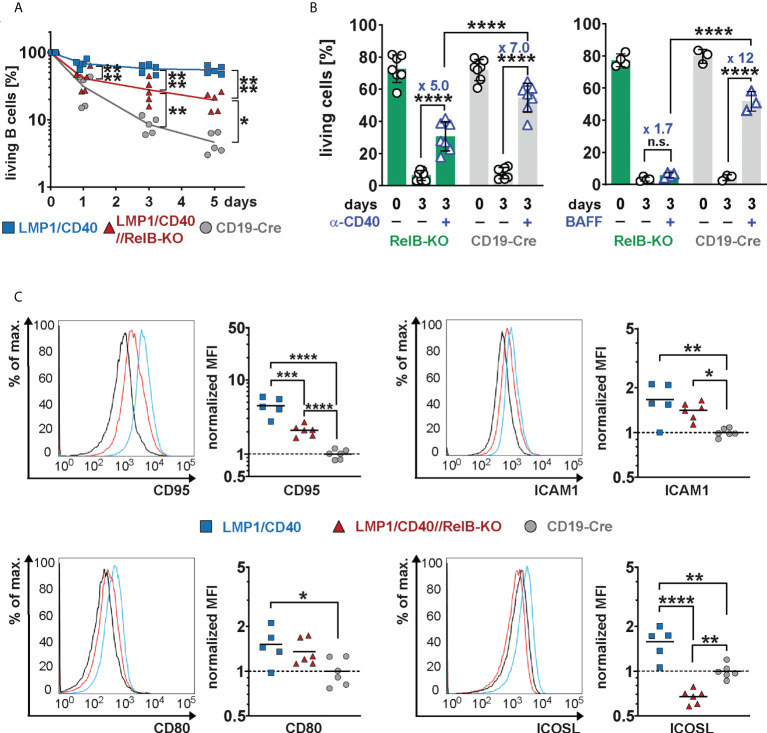
Impaired survival and activation of CD40-stimulated B cells in the absence of RelB: **(A)** Splenic B cells from mice with the indicated genotypes were cultured for up to 5 days *in vitro*. On days 0, 3 and 5, the percentage of live cells (TO-PRO-3^−^) was determined by FACS. *N*=5 independent experiments. An ordinary two-way ANOVA, Tukey´s multiple comparisons test was performed. **(B)** Splenic B cells from the indicated genotypes were stimulated for 3 days with an agonistic anti-CD40 antibody or BAFF or were kept unstimulated. Indicated are the percentages of live cells (TO-PRO-3^−^). The graph compiles the percentages of live cells on day 0 and day 3 from *N*=7 (anti-CD40) and *N≥3* (BAFF) independent experiments. Statistical analysis was performed with an ordinary two-way ANOVA, Sidak´s multiple comparisons test. **(C)** Surface expression of the indicated activation markers was determined by FACS. For each marker, one representative histogram overlay is shown. The histograms are pre-gated on live lymphocytes and CD19^+^ cells. The graphs summarize the mean fluorescence intensities (MFI) of the indicated surface markers in the indicated genotypes from N≥5 independent experiments. The average of the MFI of the controls was used to normalize each sample. Due to their log normal distribution, values were logarithmized before calculating the statistics with an ordinary one-way ANOVA with Tukey’s multiple comparison. P-values in **(A–C)** * P<0.05, ** P<0.01, *** P<0.001, **** P<0.0001. **(B, C)** Symbols represent values from individual mice, and the lines or bars indicate means.

In the absence of RelB, LMP1/CD40-expressing B cells still survived better than control B cells, but had a clear survival disadvantage compared to LMP1/CD40//RelB-proficient B cells (**Figure 4A**). Similarly, exogenous CD40-stimulation improved the survival of RelB-KO B cells, albeit not as well as that of control B cells (**Figure 4B**). Interestingly, BAFF-R stimulation, which mediates B cell survival mainly by activating the non-canonical NF-ĸB signaling pathway (42), failed to rescue RelB-deficient B cells from cell death (**Figure 4B**). These data suggest, that the survival advantage of CD40-stimulated RelB-deficient B cells is mediated by activation of the canonical NF-ĸB pathway. Additionally, inactivation of RelB in LMP1/CD40 mice led to a lower expression of the surface markers ICOSL and CD95, whereas ICAM1 and CD80 had comparable expression levels (**Figure 4C**). ICOSL has been described as being a specific target of the non-canonical NF-ĸB signaling pathway (43), which may explain its lower expression in LMP1/CD40//RelB-KO B cells than in control B cells. These data suggest that inactivation of RelB impairs the survival and activation of long-term CD40-stimulated B cells.

### RelB regulates genes involved in migration, proliferation, activation, and cytokine signaling

To get an insight into RelB-regulated genes in the situation of constitutive active CD40-signaling, we performed an RNA-Seq analysis comparing the transcriptional profile of LMP1/CD40 and LMP1/CD40//RelB-KO splenic B cells.

**Figure 5:**
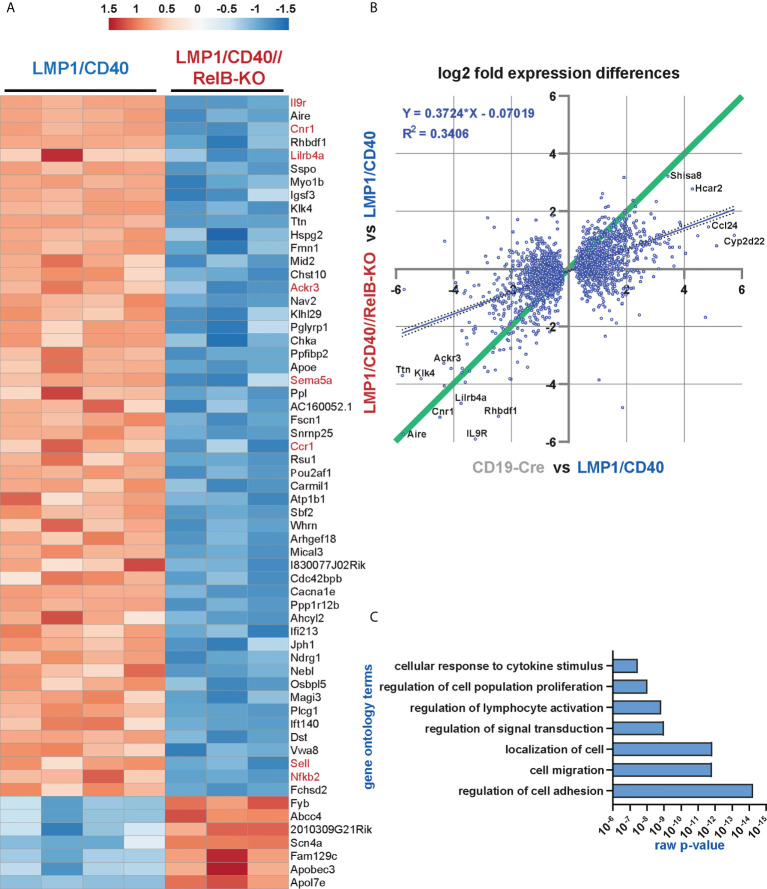
The non-canonical NF-ĸB pathway regulates genes involved in B cell survival, migration and cytokine signaling: RNA-Seq analysis was performed in two rounds. In round 1, the RNA expression profile of splenic B cells from *N*=4 LMP1/CD40 and *N*=3 LMP1/CD40//RelB-KO mice was compared, whereas in round 2, the differences in the expression profiles of *N=4* LMP1/CD40 and *N=4* CD19-Cre mice were examined. **(A)** The heat map shows the most significant differentially expressed genes between RelB-deficient and proficient LMP1/CD40 expressing B cells. The genes indicated in red were further analyzed. **(B)** Comparison of differentially expressed genes between RelB-proficient vs deficient LMP1/CD40 cells and LMP1/CD40 vs CD19-Cre cells. log2-fold differential gene expression values of all commonly expressed genes of both RNAseq analyses are plotted. A cut off at padj < 0.05 for differentially expressed genes was applied to the data from the LMP1/CD40 vs CD19-Cre analysis. Top hits are additionally marked with gene names. The resulting 2670 shared genes strongly correlated (p<0.0001) in differential expression values, the R2 value of the fitted linear regression trendline (blue, with 95% confidence intervals dotted) was added. Genes located along the green line show similar log2-fold expression differences in both RNAseq analyses. Strikingly, RelB-dependent genes with the highest log2-fold changes are located along this diagonal. **(C)** A gene ontology overrepresentation test was performed with the top 528 differentially regulated genes between LMP1/CD40 and LMP1/CD40//RelB-KO mice (from Suppl. Excel Table 1) using the online tool at [http://pantherdb.org/]. In the graph, a selection of the most significantly overrepresented GO terms for biological processes is depicted.

The heat-map shows the most significant differentially expressed genes between LMP1/CD40 and LMP1/CD40//RelB-KO B cells (**Figure 5A**). The complete list of differentially expressed genes is shown in **Supplementary Excel Table 1**. In addition, we performed a second RNA-Seq-analysis comparing the transcriptome of CD19-Cre (control) and LMP1/CD40 mice. Differentially expressed genes from both RNAseq analyses were plotted 2-dimensionally with their log2-fold changes to determine a correlation of gene expression (**Figure 5B**). The genes located along the green line (slope = 1) have similar log2 fold expression differences in both RNA-Seq analyses, suggesting that these CD40-modulated genes are mainly or even exclusively regulated by RelB. The main part of the CD40-regulated genes is not or only partially dependent on RelB, indicated by the blue regression line with a slope of 0.37. To validate our RNA-Seq analysis, we examined the RNA-expression values of individual genes whose protein expression data were already available to us. CD21 (*Cr2*), NFKB2, CD95 (*Fas*), CD80 and ICOSL (*Icosl*) were clearly upregulated in LMP1/CD40 in comparison to CD19-Cre B cells. The only discrepancy was ICAM1 (*Icam1*), which was upregulated on the cell surface but downregulated on the RNA-level in LMP1/CD40 B cells in comparison to controls. Comparing the gene expression in LMP1/CD40 vs LMP1/CD40//RelB-KO B cells revealed, that CD21 (*Cr2*), NF-ĸB2 (*Nfkb2*), CD95 (*Fas*) and ICOSL (*Icosl*) were downregulated whereas ICAM1 (*Icam1*) and CD80 (*Cd80*) had similar expression levels between LMP1/CD40//RelB-KO and LMP1/CD40 B cells (**Supplementary Figure 8A** and **Supplementary Excel file 1**). Thus, the RNA-Seq analysis was in accord with our protein expression data shown in **Figures 3C**, **4C**, and **Supplementary Figure 5A**. Since inactivation of TRAF3 and TRAF2 results in hyperactivation of the non-canonical NF-ĸB pathway (19, 44), we sought to compare the genes differentially expressed after inactivation of TRAF2 or TRAF3 with those differentially expressed in LMP1/CD40-expressing B cells with and without RelB. To this end, we generated gene sets from the differentially regulated genes between TRAF2KO or TRAF3KO and control B cells (**Supplementary Excel file 2**). A gene set enrichment analysis (GSEA) revealed that genes differentially regulated in TRAF2KO or TRAF3KO compared to WT B cells are high significantly enriched in the ranked list of all differentially expressed genes between LMP1/CD40 and LMP1/CD40//RelB-KO mice (**Supplementary Figure 8B**), further confirming that inactivation of RelB reduces the activity of non-canonical NF-ĸB-signaling. To identify the biological processes, which may explain the phenotypic differences between B cells from LMP1/CD40 and LMP1/CD40//RelB-KO mice we performed a Gene Ontology Overexpression Test with the differentially expressed genes. Amongst others, the following biological processes were identified: cell adhesion and migration; leukocyte activation and proliferation; responses to cytokines (**Figure 5C** and **Supplementary Excel file 3**). These terms reflected nicely the already observed phenotypes of RelB inactivation in LMP1/CD40-expressing B cells.

**Figure 6:**
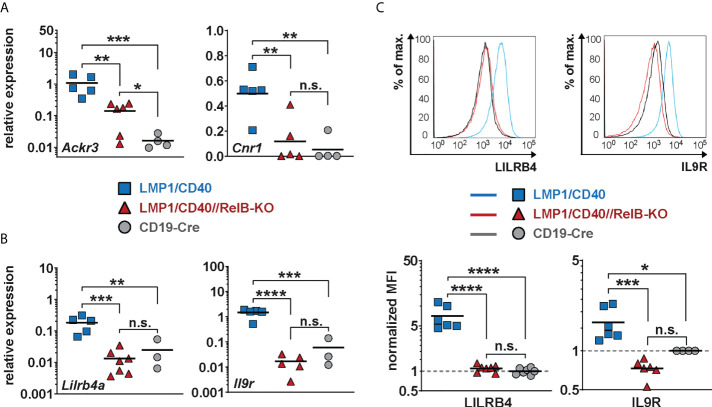
Lilrb4a and Il9r are differentially expressed at the RNA and protein level in RelB-proficient in comparison to RelB-deficient LMP1/CD40 expressing B cells: **(A, B)** The amount of mRNA of the indicated genes was determined in splenic B cells from LMP1/CD40 (blue), LMP1/CD40//RelB-KO (red) and control (CD19-Cre, grey) mice by qRT-PCR. Due to their log normal distribution, expression values of *Ackr3*, *Lilrb4a* and *Il-9R* were logarithmized before statistical analysis by an ordinary one-way ANOVA, Tukey´s multiple comparisons test. *Ackr3 N*≥4; Cnr1 N≥4; *Lilrb4a N*≥3; *IL-9R* N≥3 independent RNA preparations. **(C)** The histogram overlays show the surface expression of LILRB4 and IL-9R in the indicated genotypes. The histograms are pre-gated on live lymphocytes and CD19^+^ cells. The graphs give the summary of MFIs from independent experiments. LILRB4 *N*≥6; IL-9R N≥4. Ordinary one-way ANOVA with Tukey’s multiple comparison was performed. **(A–C)** P-values in * P<0.05, ** P<0.01, *** P<0.001, **** P<0.0001. Symbols represent values from individual mice, and the lines indicate means. n.s. = not significant.

As shown above, our data suggest that constitutively active CD40 signaling in B cells results in enhanced retention of B cells in the B cell follicles, which is relieved after inactivation of RelB. A more detailed search for genes that might influence the RelB-dependent migration and adhesion of LMP1/CD40-expressing B cells mice revealed some interesting candidates, such as *Itgax* and *Sell* (L-Selectin, CD62-L), *Ccr1* and *Ccr6* (encoding for chemokine receptors), *Sema3b* and *Sema5a* (encoding for Semaphorines) and *S1pr1* (encoding the sphingosine-1-phosphate-receptor1) (**Supplementary Figure 9**). Interestingly, S1PR1, which guides MZB cells into the MZ was strongly downregulated in LMP1/CD40 in comparison to control B cells, whereas this repression was released after inactivation of RelB. Among the top-most differentially-regulated genes were *Ackr3* and *Cnr1* (**Figure 5A**). *Ackr3* (CXCR7) is an atypical chemokine receptor that inhibits the migratory capacity of B cells towards CXCL12, which is highly expressed on BM stromal cells, in the splenic red pulp and in marginal zone bridging channels (45–47). *Cnr1*, encoding for a cannabinoid receptor, has been suggested to retain Mantle Cell Lymphomas in the B cells follicles (48). Thus, upregulation of these two genes in the LMP1/CD40 B cells may at least partially explain their enhanced retention in the B cell follicles. We further confirmed the RelB-dependent upregulation of *Ackr3* and *Cnr1* by qRT-PCR (**Figure 6A**).

Additionally, we found *Lilrb4a* and *Il9r* among the top-most differentially-regulated genes (**Figure 5A**). Their downregulation after inactivation of RelB was of keen interest to us, as both genes are overexpressed in some B cell lymphomas. LILRB4 has been suggested to play a role in immunosurveillance while IL-9R signaling appears to have a positive influence on B cell proliferation and survival (49–51). Thus, the upregulation of these genes may have a direct influence on lymphomagenesis. This prompted us to study the regulation of these two genes in more detail. We confirmed the RelB-dependent upregulation of *Lilrb4* and *Il9r* in LMP1/CD40-expressing B cells by qRT-PCR (**Figure 6B**) and at the protein level by FACS (**Figure 6C**).

**Figure 7:**
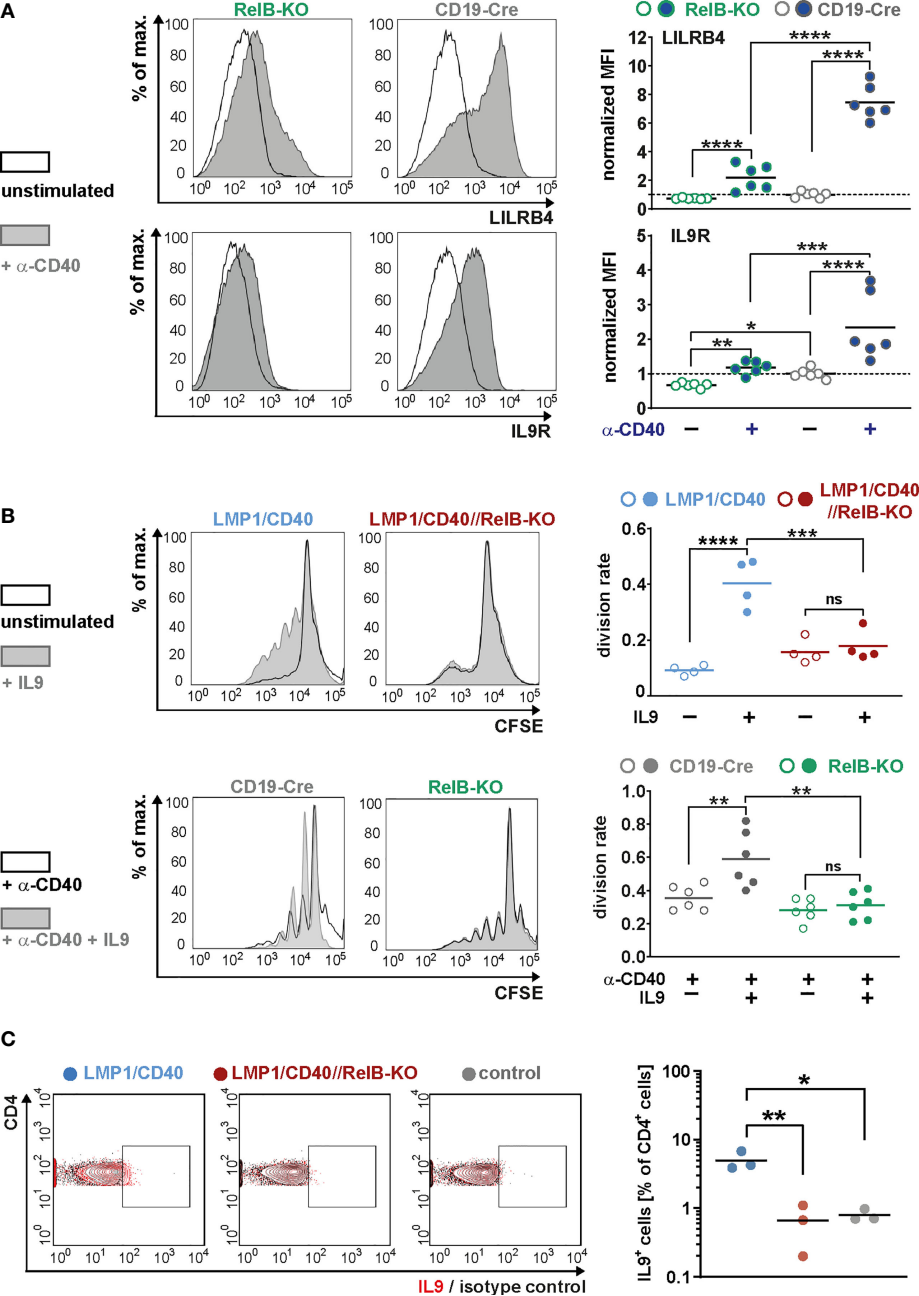
RelB-dependent upregulation of LILRB4 and IL-9R upon CD40-stimulation: **(A)** MACS purified B cells were cultured in the presence of an agonistic anti-CD40 antibody or were left unstimulated for two days. The surface expression of LILRB4 and IL-9R was determined by FACS. Representative histogram overlays of unstimulated and CD40-stimulated B cells from RelB-KO and CD19-Cre control mice are shown. The histograms are pre-gated on live cells. The graphs compile the values of *N*=6 independent experiments. For normalization, the individual values were divided by the average of the unstimulated controls. Due to their log-normal distributions, values were logarithmically transformed before statistical analysis with an ordinary two-way ANOVA, Tukey´s multiple comparisons test. **(B)** The proliferation of splenic B cells isolated from mice with the indicated genotypes was analyzed after CSFE staining. In the upper row, splenic B cells were cultured with and without IL9 stimulation for three days. In the lower row, cells were additionally stimulated with CD40. Representative histogram overlays of the CFSE staining of stimulated and unstimulated B cells from the indicated genotypes are shown. The histograms are pre-gated on live cells. The graphs compile the division rates in the individual samples from *N*=4 (LMP1/CD40; LMP1/CD40//RelB-KO) and *N*=6 (RelB-KO; CD19-Cre) mice. Statistics were calculated by an ordinary two-way ANOVA, Tukey´s multiple comparisons test. **(C)** Representative FACS plot for the detection of IL-9 producing T cells. The FACS plot shows an overlay of CD4^+^ T cells stained with an anti-IL-9 antibody (red) or the corresponding isotype control (black) in the indicated genotypes (control: CD19-Cre). The gates for IL-9 positive cells was set according to the isotype control. The graph compiles the percentages of IL-9^+^CD4^+^ T cells from *N*=3 independent experiments. Ordinary one-way ANOVA with Tukey’s multiple comparison was performed. **(A–C)** p-values are indicated as * P<0.05, ** P<0.01, *** P<0.001, **** P<0.0001. Dots represent values from individual mice, and the lines indicate means. n.s. = not significant.

Moreover, we could show that RelB is responsible for most of the upregulation of these two genes by exogenous CD40-stimulation (**Figure 7A**). Higher IL-9R expression on the cell surface of LMP1/CD40-expressing (**Figure 6C**) and CD40-stimulated RelB-proficient B cells (**Figure 7A**) was correlated with an enhanced proliferation after IL9 stimulation compared to their RelB-deficient counterparts (**Figure 7B**). In contrast, RelB-proficient and RelB-deficient LMP1/CD40-expressing B cells proliferated comparably in the presence of IL-4. Simultaneous stimulation with IL-9 and IL-4 enhanced the proliferation of LMP1/CD40, but not of LMP1/CD40//RelB-KO B cells (**Supplementary Figure 10**). These data showed that LMP1/CD40//RelB-KO B cells do not have a general proliferation defect, but only an IL-9 dependent proliferation impairment. IL-9 has been described to be secreted by CD4^+^ T cells - the so-called Th9 cells (52, 53). Interestingly, we also detected a higher percentage of IL-9^+^ CD4^+^ T cells in LMP1/CD40 in comparison to LMP1/CD40//RelB-KO mice (**Figure 7C**). Additionally, we found slightly, but significantly, higher expression of OX40-L on the surface of B cells from LMP1/CD40 in comparison with LMP1/CD40//RelB-KO mice (**Supplementary Figure 11**). Since OX40 signaling has been shown to favor the generation of Th9 cells (54) this could at least partially explain the higher frequency of IL9-expressing CD4^+^ T cells in LMP1/CD40 as compared with LMP1/CD40//RelB-KO mice. These data suggest that continuous CD40 stimulation and consequent constitutive activation of the non-canonical NF-ĸB signaling in B cells triggers an autoregulatory loop, resulting in upregulation of the IL-9R on B cells and attraction and/or generation of IL-9 producing T cells. Thus, it is tempting to speculate that RelB-proficient LMP1/CD40 expressing B cells are continuously stimulated by IL-9 *in vivo*, which may contribute to their expansion.

### The non-canonical NF-κB signaling pathway enhances the incidence of B cell lymphomas

**Figure 8:**
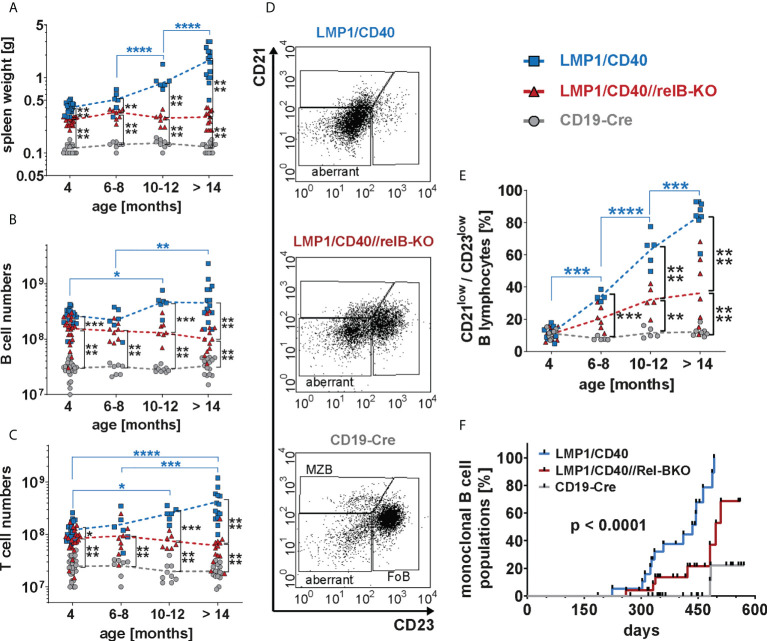
Inactivation of RelB reduces the lymphoma incidence in LMP1/CD40 mice: **(A)** The graph depicts the splenic weights of LMP1/CD40, LMP1/CD40//RelB-KO and CD19-Cre control mice at different ages. **(B, C)** Splenic B and T cell numbers of mice with the indicated genotypes with different ages. **(A–C)** Values were log-normal distributed and were therefore logarithmized before statistics was calculated with an ordinary two-way ANOVA, Tukey´s multiple comparisons test *N*≥6 **(D)** Representative FACS plots of the splenic CD21/CD23 B cell populations from aged mice with the indicated genotypes. The mice were at an approximate age of 1 year. FACS plots are pre-gated on live lymphocytes and CD19^+^ B cells. **(E)** The graph compiles the percentages of the CD21^low^CD23^low^ B cell population in mice with the genotypes and age as indicated. An ordinary two-way ANOVA, Tukey´s multiple comparisons test was used in statistical analysis. *N*≥4. **(A–C, E)** P values are indicated as * P<0.05, ** P<0.01, *** P<0.001, **** P<0.0001. Symbols represent values from individual mice, and the lines indicate means. **(F)** Monoclonal B cell populations were determined by Southern blot analysis using a radioactive J_H_ probe as shown in (Supplementary Figure 14). The graph compiles the results of individual mice. Black dots indicate individual mice analyzed at the indicated time point. Mice were analyzed when they showed signs of disease, had a clearly palpatable splenomegaly or reached an age of 19 months. For statistical analysis a log-rank (Mantle Cox) test was performed.

Finally, we analyzed whether RelB-deficient LMP1/CD40 mice still develop lymphomas. Cohorts of LMP1/CD40- and LMP1/CD40//RelB-KO mice as well as control mice were analyzed at the ages of 6-8 months, 10-12 months and older than 14 months. We stopped our observation at 18 months. The splenic weight, as well as B- and T cell numbers increased considerably during the course of observation in LMP1/CD40 mice, but stayed rather stable in LMP1/CD40//RelB-KO and control mice (**Figures 8A–C**). The increase in T cell numbers in LMP1/CD40 mice was mainly due to an expansion of CD4^+^ T cells, whereas CD8^+^ T cell numbers in LMP1/CD40 and LMP1/CD40-RelB-KO mice remained relatively similar during aging (**Supplementary Figure 12**). During this study period, FACS analysis revealed the appearance of an aberrant population with the phenotype B220^low^CD19^+^CD43^+^CD21^low^CD23^low^ (**Figure 8D**). In comparison to LMP1/CD40 mice, this aberrant population occurred at a delayed time point and was less prominent in the LMP1/CD40//RelB-KO mice and almost absent in controls (**Figure 8E**). Annexin/7AAD stainings revealed that the CD21^+^CD23^+^ population was less apoptotic in LMP1/CD40 and LMP1/CD40//RelB-KO B cells in comparison to controls, without a significant difference between LMP1/CD40 and LMP1/CD40//RelB-KO B cells. However, a marked difference in the survival of LMP1/CD40 in comparison to LMP1/CD40//RelB-KO B cells was observed in the CD21^+^CD23^−^ and in particular in the CD21^−^CD23^−^ population. Compared with the significantly better surviving CD21^−^CD23^−^ population from LMP1/CD40 mice, this population appeared highly apoptotic when isolated from LMP1/CD40//RelB-KO and control B mice (**Supplementary Figure 13**). The higher apoptosis rate and cell death in the CD21^−^CD23^−^ B cells population from LMP1/CD40//RelB-KO mice in comparison to LMP1/CD40 mice may be one of the reasons that the aberrant B cell population is less expanded in RelB-deficient in comparison to RelB-proficient LMP1/CD40 mice.

Further analysis of lymphoma development in these mice revealed that monoclonal B cell populations occurred earlier and with a higher incidence in LMP1/CD40 mice in comparison to LMP1/CD40//RelB-KO mice (**Figure 8F** and **Supplementary Figure 14**). Histological sections of diseased LMP/CD40 and LMP1/CD40//RelB-KO mice displayed comparable histopathological alterations with an expanded White Pulp (WP) area. The WP showed a loss of its typical architecture with high numbers of B220^+^ cells in the periphery of the expanded WP area and in the Red Pulp (RP). CD3^+^ lymphocytes were scattered within the WP in abundant numbers. A few Ki-67^+^ cells were present in the WP, the majority of Ki-67^+^ cells being localized in the RP (**Supplementary Figure 15**). These data suggest that lymphomas still arise in LMP1/CD40//RelB-KO mice. However, in comparison to LMP1/CD40 mice, the incidence of monoclonal B cell expansions was lower and occurred with a longer latency period. Thus, our data suggest that continuous activation of non-canonical NF-ĸB signaling in long-term CD40-stimulated B cells promotes the transition from pre-malignant B cell expansions to monoclonal B cell lymphomas.

## Discussion

Several human B cell malignancies exhibit constitutive activation of the non-canonical NF-ĸB pathway (12). In addition, mouse mutants with hyperactive non-canonical NF-ĸB signaling in B cells develop B cell expansions and lymphomas (10, 20, 22, 27, 44). This suggests that aberrant activation of non-canonical NF-ĸB signaling drives B cell lymphoma development. Nevertheless, the contribution of RelB to pre-malignant B cell expansions and initiation of lymphomagenesis is largely unknown.

To address this issue, we inactivated RelB in LMP1/CD40 mice (27). These mice express a fusion protein consisting of the transmembrane domain of LMP1 and the C-terminal signaling part of CD40 (LMP1/CD40) resulting in constitutively active CD40-signaling in B cells. In premalignant B cells, LMP1/CD40-expression activates the MAPK Erk and Jnk as well as the non-canonical NF-ĸB signaling, suggesting that long-term CD40-stimulation results in the preferential activation of the non-canonical NF-ĸB signaling and not as much of the canonical one. A similar NF-ĸB profile like in pre-malignant LMP1/CD40 B cells was described in various HL cell lines, which also had increased p52, p50 and RelB levels, but rather normal levels of RelA in the nucleus (27, 55, 56). Since HL cells express high levels of CD40, it is likely that they are continuously stimulated by ligand expressing cells from the tumor microenvironment and thus receive a long-lasting CD40-signal similar to LMP1/CD40-expressing B cells (57).

Inactivation of RelB resulted in a large reduction of p100 in B cells, which may be a consequence of both reduced transcription (as revealed by our RNA-Seq analysis) and reduced stability (58). Although the remaining p100 levels were nearly completely processed in RelB-KO B cells, p52 levels were clearly lower than in wildtype B cells. De Silva and colleagues demonstrated that B cell numbers in RelB/NF-ĸB2 double deficient B cells decreased much stronger than in single RelB-KO mice (6). These data indicate that the residual amounts of p52 are sufficient to rescue RelB-deficient B cells *in vivo*, highlighting the importance of p52/c-Rel or p52/RelA or even p50/p52 dimers for the homeostasis of B cells *in vivo*. P100 levels were also reduced in LMP1/CD40//RelB-KO in comparison to LMP1/CD40 and control B cells. The reduced p100 levels resulted in significantly lower p52 levels in LMP1/CD40//RelB-KO in comparison to LMP1/CD40 B cells, which were now comparable to those of control B cells. These data suggest that after inactivation of RelB non-canonical NF-ĸB signaling was also diminished with respect to p52 at control levels. Attenuated non-canonical NF-ĸB signaling in LMP1/CD40//RelB-KO in comparison to LMP1/CD40 cells is also reflected in the strong enrichment of TRAF2 and TRAF3 KO gene sets in the GSEA with our differentially regulated genes from RelB-proficient vs -deficient LMP1/CD40-expressing B cells. However, the residual amounts of p52 in LMP1/CD40//RelB-KO B cells may still complex with p65 or c-Rel and thereby contribute to the activity of NF-ĸB signaling. Thus, inactivation of both RelB and p100, resulting in complete inactivation of non-canonical NF-ĸB signaling might have a stronger effect on the expansion of LMP1/CD40-expressing B cells than the single inactivation of RelB. Nevertheless, our approach has the advantage that we can learn how RelB contributes to gene regulation and lymphomagenesis. B cells from LMP1/CD40 mice mainly accumulated in the spleen and the LN, whereas they were reduced in the blood and BM in comparison to controls. Moreover, even the MZB cells from LMP1/CD40 mice appeared to selectively reside in the follicles, instead of migrating into the MZ. All these phenotypes were reverted in LMP1/CD40//RelB-KO mice. These data suggest that hyperactivation of the non-canonical NF-ĸB pathway enhances the retention of B cells in the follicles of SLO. Our RNA-Seq analysis revealed several RelB-regulated genes, which may be responsible for this phenotype. For example, the Sphingosine-1-phosphate receptor 1 (*S1pr1*), which guides B cells into the MZ (59), was downregulated in LMP1/CD40 in comparison to control B cells and had a lower expression in B cells of LMP1/CD40 compared to LMP1/CD40//RelB-KO mice. In contrast, *Ackr3*, encoding for the atypical chemokine receptor ACKR3/CXCR7, was higher expressed in B cells from LMP1/CD40 than from LMP1/CD40//RelB-KO and control mice. ACKR3 binds to the chemokine ligand CXCL12 with a higher affinity than CXCR4 without initiating signaling and thus prevents CXCR4-mediated migration (60). CXCR4-deficient mice show enhanced migration of B cells into splenic follicles, a reduced retention of B cells in the BM and reduction of B1 cells in the peritoneal cavity (61). In contrast, homing of ACKR3-KO B cells to the spleen is reduced (62). Hence, decreased CXCR4 sensitivity caused by the upregulated ACKR3 expression may explain most of the phenotype differences in the migratory behavior of B cells between LMP1/CD40 and LMP1/CD40//RelB-KO mice. Yet another candidate gene, which could entrap LMP1/CD40-expressing B cells in the follicles, is the cannabinoid receptor 1 (CNR1). Normal lymphocytes express low levels of CNR1, but CNR1-expression is upregulated by CD40-stimulation (63), as well as by LMP1/CD40-expression as shown here. Interestingly, CNR1 expression is associated with the retention of MCL cells in the mantle zone of the B cell follicles, whereas leukemic forms of MCL lose their CNR1-expression (48). An effect of RelB-regulated genes on the migration of B cells has been reported earlier. The group of Falk Weih reported in 2001 that the complete inactivation of RelB in mice results in a homing defect, which was mainly due to reduced expression of the chemokine ligands CXCL12 (BLC), EBI1-ligand (ELC) and CCL21 (SLC) in the spleen (5). These chemokine ligands are expressed in stromal cells, hinting to a function of RelB in non-lymphocytes. In addition, Ulf Klein’s group reported differential expression of genes involved in migration after 6 hours of BAFF or CD40 stimulation of RelB/NFĸB2 double-deficient B cells in comparison to control cells. However, the differentially expressed genes described in his analysis (6) have little overlap with genes detected in our analysis. The differences between the systems could be due to the different genetic background of the B cells (NFĸB2/RelB-KO vs. RelB-KO) or due to the difference between constitutive CD40-stimulation *in vivo* and relatively short-term CD40- or BAFF-stimulation of *ex vivo* isolated B cells. In particular, the strong activation of the canonical NF-ĸB pathway that occurs upon CD40 stimulation *in vitro* might make it difficult to filter out the contribution of non-canonical NF-ĸB transcription factors to gene regulation. One example is the clear difference, which we found in the expression of ICOSL and CD95 in LMP1/CD40- compared with LMP1/CD40-RelB-KO B cells (**Figure 4**) but not after CD40-stimulation of *ex vivo* isolated RelB-KO and control B cells (**Supplementary Figure 16**).

Another interesting gene that was differentially expressed in RelB-proficient and -deficient LMP1/CD40-expressing B cells was *Lilrb4.* LILRB4 is not expressed on healthy B cells, but can be detected on CD5^+^ neoplastic B cells of CLL patients (51, 64). LILRB4 signaling has been suggested to suppress T cell responses (50, 65–67). Hence, upregulation of LILRB4 in long-term CD40-stimulated B cells could contribute to lymphomagenesis by enhancing immune evasion. One of the top-most differentially-regulated genes, which was contained in the GO term “cytokine signaling” was *Il9r*. Physiologically, IL-9R signaling plays a role in the differentiation of memory B cells and their humoral recall response by facilitating proliferation before memory B cells differentiate into plasma cells (68, 69). Interestingly, overexpression of the IL-9R has been found in some DLBCL, and further experiments have indicated its potential role in the survival, proliferation, and drug resistance of DLBCL (49, 70). Additionally, IL9 promotes the expansion of a B1 cell like population (71), which is the (pre) malignant B cell type in LMP1/CD40 mice. We found that in LMP1/CD40 mice next to the IL-9R upregulation in B cells, there is also an increase in the proportion of IL9-secreting CD4^+^ T cells. These data suggest that constitutive CD40-stimulation and consequently hyperactivation of the non-canonical NF-ĸB pathway in B cells has an impact on T cells as also clearly evidenced by higher T cell expansion in LMP1/CD40 in comparison to control mice. Thereby activation of the non-canonical NF-ĸB signaling pathway appears to activate in particular CD4^+^ T cells, which increase more in LMP1/CD40 than in LMP1/CD40//RelB-KO mice during aging. The higher expression of ICOSL, and OX40-L on the surface of B cells from LMP1/CD40 as compared to LMP1/CD40//RelB-KO and control mice may explain, at least in part, the higher expansion of CD4^+^ T cells during aging and the higher percentage of CD4^+^IL-9 secreting T cells in LMP1/CD40 mice.

In LMP1/CD40 mice, lymphoma incidence and expansion was reduced in the absence of RelB. Recently, a new subgroup of DLBCL with activated RelB has been described (17). These DLBCL subtypes significantly correlated with inactivating TRAF3 and TRAF2 mutations. Assuming that in humans TRAF2 and TRAF3 mutations occur in premalignant cells, they may contribute to the initiation of B cell lymphomas through a similar RelB-dependent mechanism as we describe here for LMP1/CD40 mice. We have previously found, that the pattern of signal activation is highly consistent in premalignant B cells from LMP1/CD40 mice, but varies considerably in lymphomas arising in different mice (including activation of the canonical NF-ĸB pathway in some lymphomas) (27). This suggests that the acquisition of secondary mutations influences the pattern of signaling pathways, which are finally activated in lymphoma cells (21). The same could happen in the transition from premalignant to malignant cells in humans, explaining why in DLBCL with TRAF2/3 mutations the canonical NF-ĸB pathway is often coactivated with the non-canonical one.

In sum, our data suggest that RelB accelerates the initiation and progression of B cell lymphomas by regulating genes involved in cell migration, proliferation and cytokine signaling. However, our data also show that RelB is not absolutely required for tumorigenesis suggesting that RelB-deficiency can be compensated by activation of other signaling pathways such as activation of canonical NF-кB signaling.

## Data availability statement

The datasets presented in this study can be found in online repositories. The names of the repository/repositories and accession number(s) can be found below: NCBI GEO under BioProject ID PRJNA823508.

## Ethics statement

The animal study was reviewed and approved by institutional committee on animal experimentation and the Government of Upper-Bavaria.

## Author contributions

Contribution: LK, SV, KS, TB: performed research, interpreted data, YW, UR performed research, LJS quantified WB for NF-ĸB analysis, LK helped to write the manuscript; DS, SG, DJR performed bioinformatic analyses; DJR edited the manuscript. HB performed RNA-sequencing; AF, AB performed and interpreted the histology of paraffin-sections; FW provided mice and discussed and interpreted data; DK provided essential reagents; RR discussed and interpreted data; LJS analyzed and interpreted data, prepared figures; UZ-S designed experiments, interpreted data, wrote the manuscript. All authors contributed to the article and approved the submitted version.

## Funding

This work was supported by grants from the Deutsche Forschungsgemeinschaft (SFB1243 TPA13, TPA16; DFG RA 1629/4-1) and the Deutsche Krebshilfe (109131, 70113859).

## Acknowledgments

We thank the animal facility of the Helmholtz Center and our animal caretakers for excellent housing of the mice and Krisztina Zeller for excellent technical assistance.

## Conflict of interest

The authors declare that the research was conducted in the absence of any commercial or financial relationships that could be construed as a potential conflict of interest.

## Publisher’s note

All claims expressed in this article are solely those of the authors and do not necessarily represent those of their affiliated organizations, or those of the publisher, the editors and the reviewers. Any product that may be evaluated in this article, or claim that may be made by its manufacturer, is not guaranteed or endorsed by the publisher.

## Abbreviations

BM, Bone marrow; MZ, Marginal zone; MZB, Marginal zone B cells; FoB, Follicular B cells; SLO, secondary lymphoid organs; WP, white pulp; RP, Red Pulp; HL, Hodgkin-Lymphoma; MCL, Mantle cell lymphoma, DLBCL, Diffuse large B cell lymphoma.
