# Supplementary material for: RelB contributes to the survival, migration and lymphomagenesis of B cells with constitutively active CD40 signaling
Source: Front Immunol. 2022 Aug 30;13:913275. doi: 10.3389/fimmu.2022.913275 (PMC9468873; doi:10.3389/fimmu.2022.913275)
Supplement: Supplementary file 1 [file DataSheet_1.pdf]

Supplementary Material

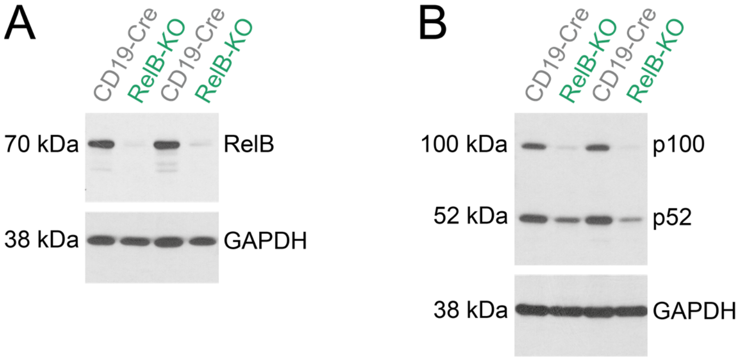

Supplementary Figure 1: Inactivation of RelB results in reduced basal levels of p100 and p52. Western blots were performed with extracts from splenic B cells isolated from RelB-KO or CD19-Cre (control) mice. Western blots were incubated with an anti-RelB antibody (A) or an antibody raised against the C-terminal part of p100 detecting both p100 and p52. GAPDH was used as the loading control. The blots were performed with two independent extract preparations.

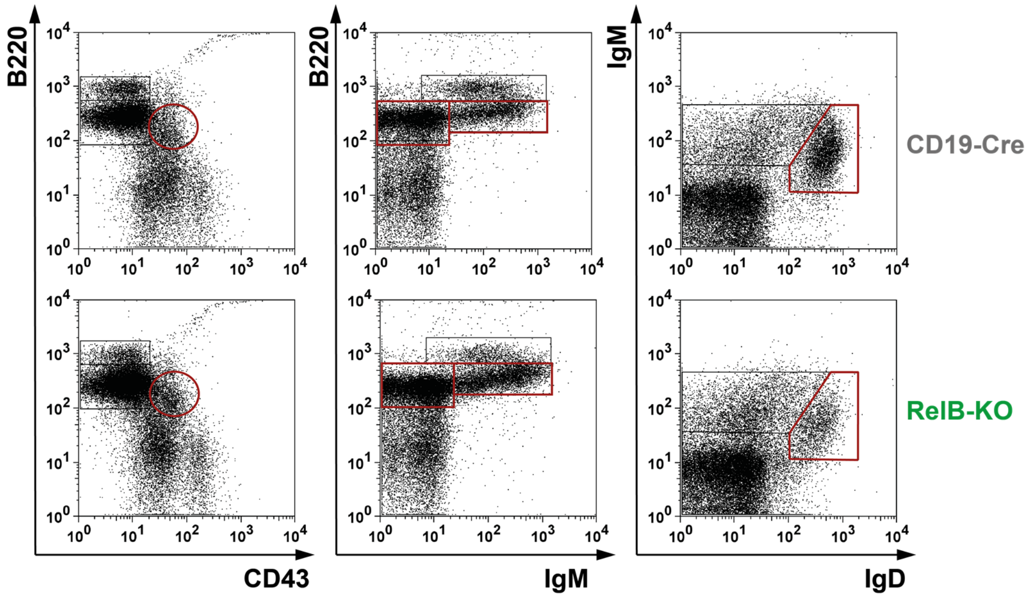

%	pro B	pro/pre B	immature	recirculating
RelB-KO	7.2±2.4	47.3±8.3	15.4±5.1	4.8±0.91 **
CD19-Cre	8.1±2	39.7±5.9	14.1±3.6	11.4±4.0

x10^6				
RelB-KO	1.0±0.3	6.3±3.2	2.2±0.8	0.71±0.17 **
CD19-Cre	1.1±0.2	5.6±2.2	1.9±0.5	1.5±0.48

Supplementary Figure 2: B cell development is normal in the BM of RelB-KO mice. Exemplary FACS plots for developing and recirculating B cells in the BM. The plots are pre-gated on live lymphocytes. Gating strategy to determine the fractions of CD43$^+$ pro (and pre-pro), IgM$^-$B220^{low} pro/pre, IgM$^+$B220^{low} immature as well as IgD$^+$IgM$^+$recirculating B cells. B220^{low}CD43$^+$ and B220^{low}IgM$^-$ gates may additionally contain some NK cell precursors and CD11c plasmacytoid DC (Nikolic et al., EJI 2002, 32: 686-692; Rolink et al., JEM, 1996, 183: 187-194). The gates that were used to calculate the percentages of the B cell populations in the BM are indicated in red. The percentages and total cell numbers of the different B cell populations in the BM are compiled in the table. Numbers represent the mean +/-SD of *N*=5 individual mice. Unpaired two tailed t-test was performed. Numbers of recirculating B cells were lognormal distributed. Therefore, these values were logarithmically transformed before statistical analyses. ** P<0.01.

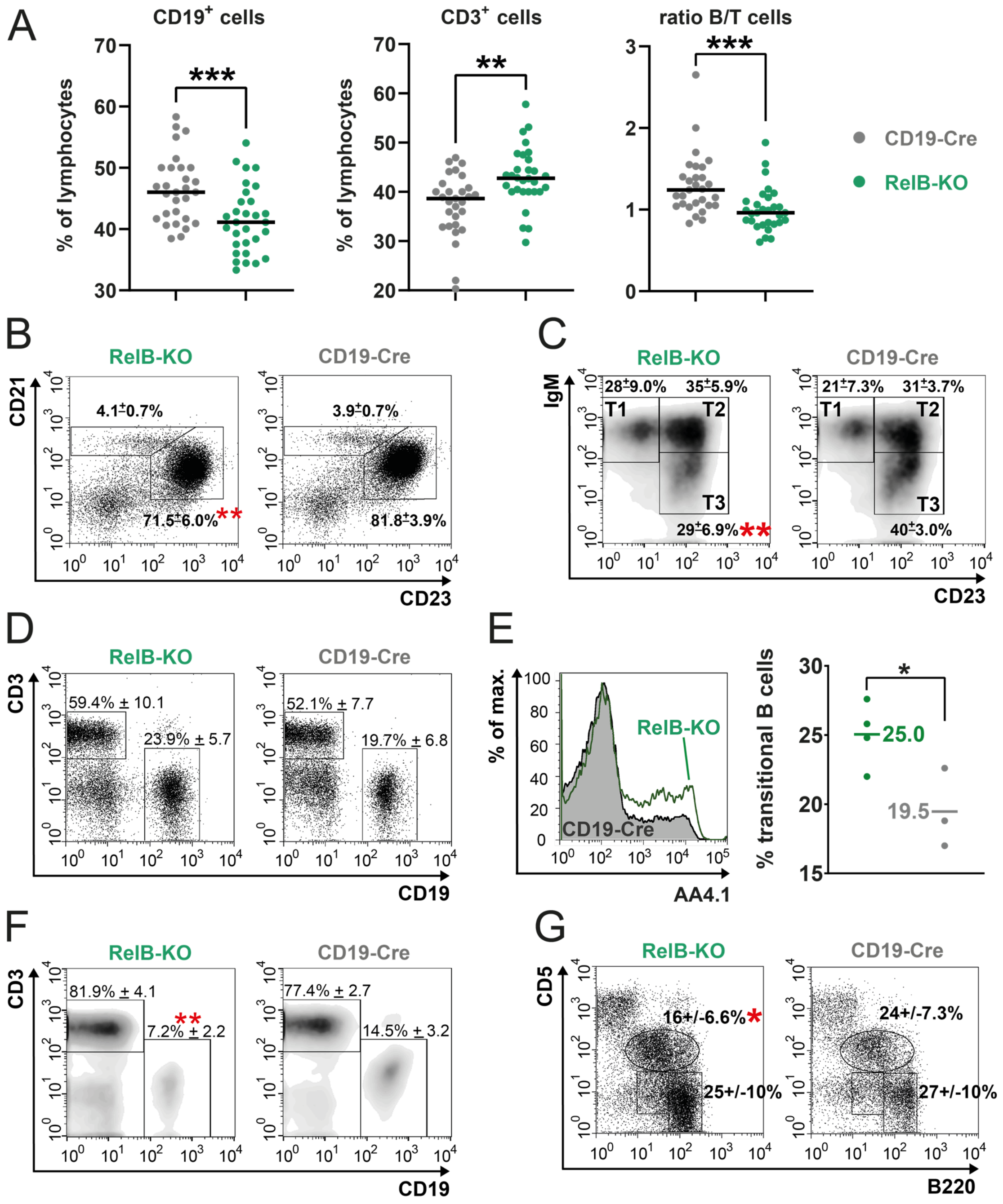

Supplementary Figure 3: RelB inactivation results in decreased numbers of B cells in secondary lymphoid organs. (**A**) The graphs summarize the percentages of B and T lymphocytes and the ratio between B and T lymphocytes in the spleen of RelB-KO in comparison to control mice (**B**) Exemplary FACS blots from splenic B cells to detect MZB ($CD21^{high}CD23^{low}$) and FoB ($CD21^{int}CD23^{high}$) cells. The plots are gated on live $B220^{+}$ lymphocytes. (**C**) Gating strategy for the detection of T1 ($IgM^{high}CD23^{-}$), T2 ($IgM^{high}CD23^{+}$) and T3 ($IgM^{low}CD23^{+}$) cells in the spleen. The plots are pre-gated on living $B220^{+}AA4.1^{+}$ lymphocytes. (**B+C**) Numbers indicate the mean +/-SD of $N \geq 5$ individual mice. (**D+E**) FACS analysis of B cells in the blood. (**D**) Cells were stained to detect T cells ($CD3^{+}$) and B cells ($CD19^{+}$). FACS plots are pregated on live TO-PRO-3^{-} lymphocytes. Numbers represent the mean +/-SD of N≥7 individual mice. (**E**) Transitional B cells were determined as $AA4.1^{+}B220^{+}$. N=3 CD19-Cre and N=4 RelB-KO. (**F**) FACS analysis with single cell suspensions from the LN to detect the percentages of B ($CD19^{+}$) and T ($CD3^{+}$) lymphocytes. Numbers represent the mean +/-SD of $N \geq 6$ individual mice. (**G**) B cell populations in the peritoneal cavity. FACS plots were pre-gated on live lymphocytes. The gating of B1a ($B220^{low}CD5^{+}$), and B2 ($B220^{+}CD5^{-}$) cells is shown. Numbers represent the mean +/-SD of $N \geq 7$ individual mice. Statistical analyses were conducted with an unpaired two-tailed t-test. * P<0.05, ** P<0.01, *** P<0.001.

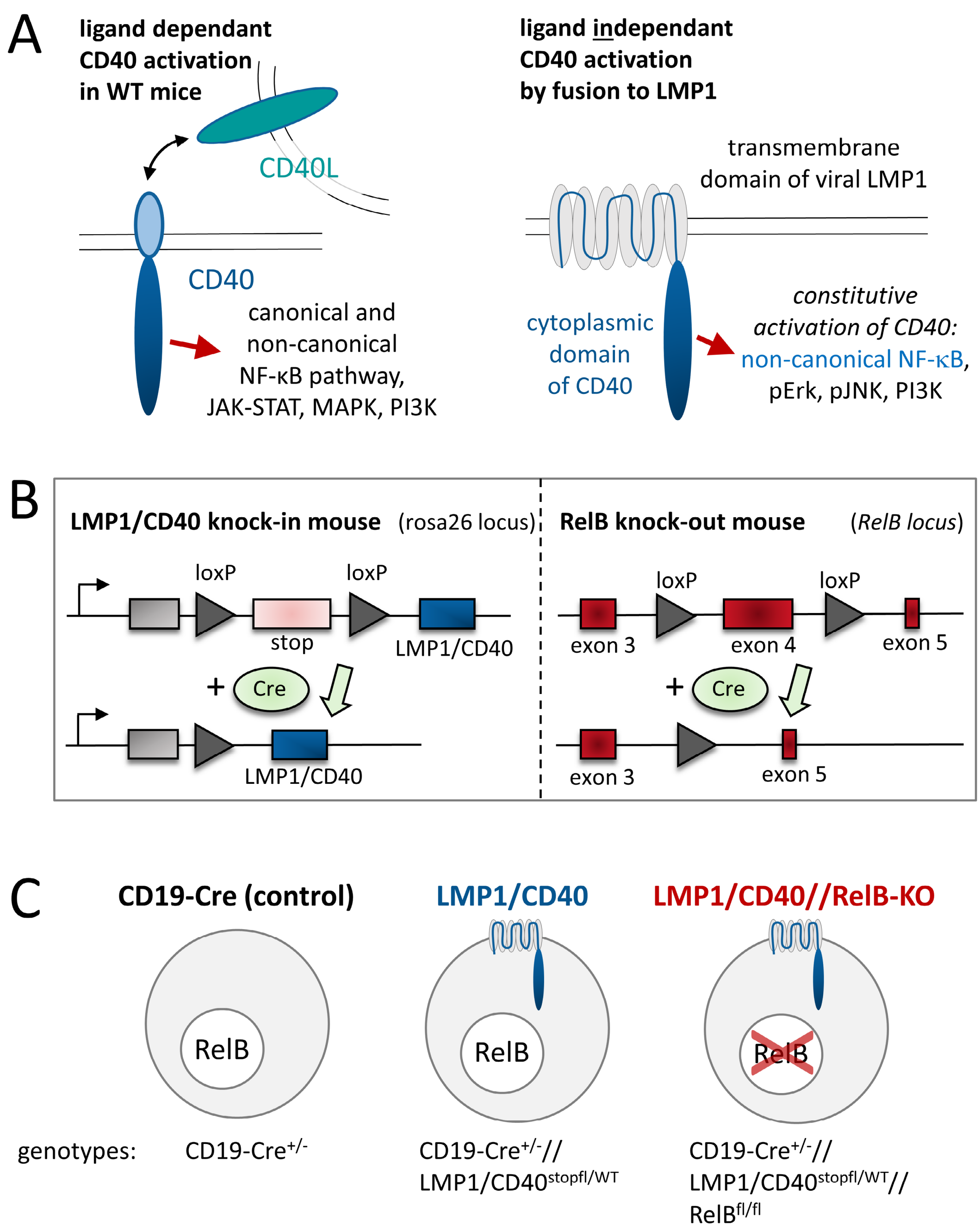

Supplementary Figure 4: Schematic presentation of the LMP1/CD40//RelB-KO mouse model. (**A**) LMP1 is an Epstein-Barr viral protein that mimics to some extent a constitutive active CD40 receptor. The constitutive activity of LMP1 is mediated by its transmembrane domain. To generate a transgene that induces a constitutive active CD40 signal instead of a LMP1 signal, we exchanged the C-terminal signaling part of LMP1 with that of CD40 (Gires et al., 1998, Hömig-Hölzel et al., 2008). (**B**) The LMP1/CD40 transgene was inserted together with a loxP flanked stop-cassette into the *rosa26*-locus (LMP1/CD40 mice). To inactivate RelB in LMP1/CD40 mice we crossed them to RelB$^{fl/fl}$ mice, containing a floxed exon 4 of RelB (Riemann et al., 2017). B cell specific activation of LMP1/CD40 and inactivation of RelB was achieved by mating LMP1/CD40$^{stopfl/WT}$//RelB$^{fl/fl}$ mice with CD19-Cre mice (LMP1/CD40//RelB-KO mice). (**C**) Graphical depiction of the protein expression in the transgenic B cells from the different genotypes.

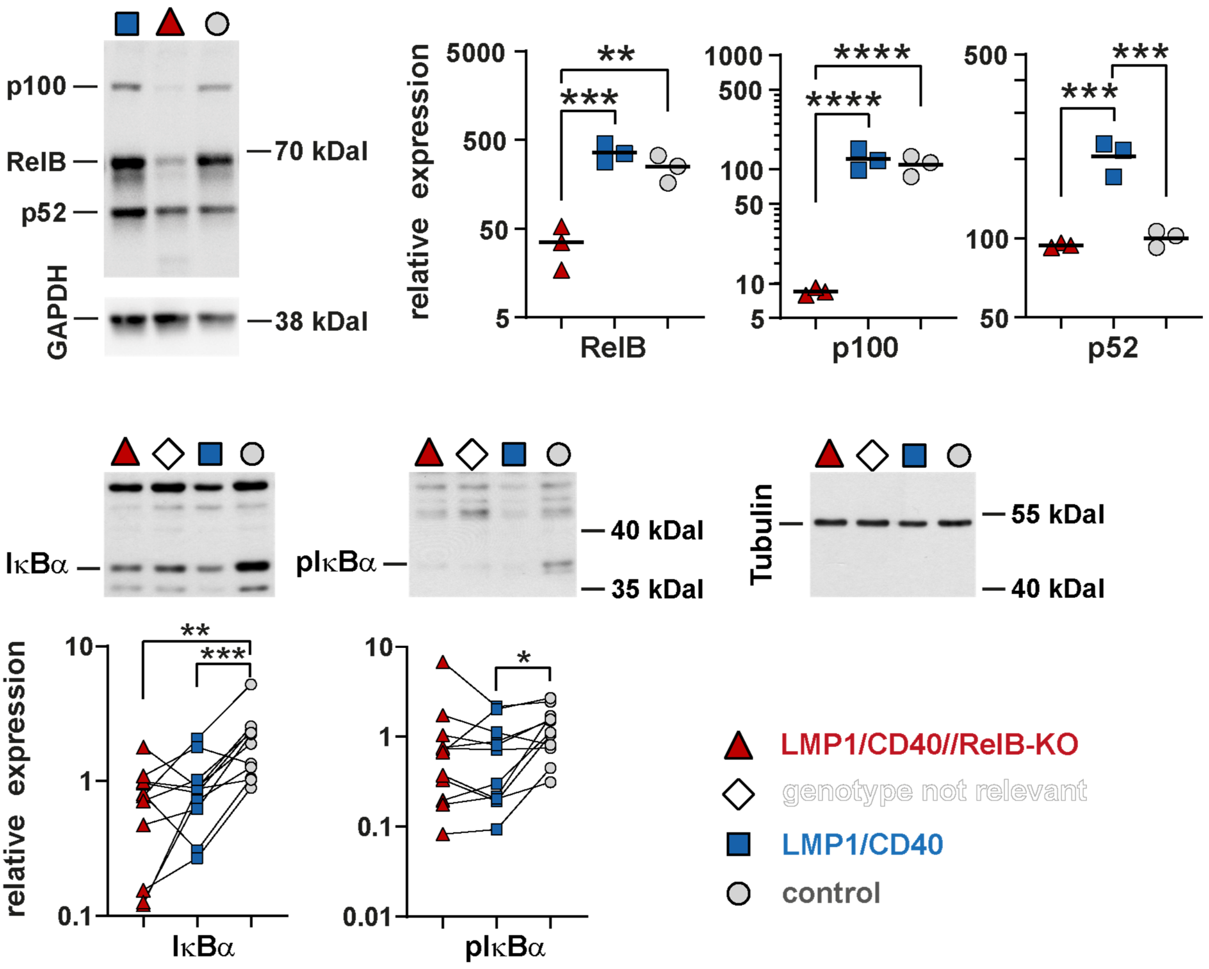

Supplementary Figure 5: p100 and p52 levels are reduced in LMP1/CD40-expressing B cells in the absence of RelB. Western blot with whole cell extracts from splenic B cells from the indicated genotypes after staining with antibodies raised against RelB and p100/p52 **(A)** as well as IκB-α and pIκB-α **(B)**. GAPDH (A) and Tubulin (B) were used as loading control and for normalization of quantified proteins. **(A)** The graphs compile the amounts of the indicated proteins from *N*=3 independent Western blots. Statistical analysis was performed after logarithmic transformation due to a lognormal distribution of the values. Ordinary one-way ANOVA with Tukey's multiple comparison was performed with ** $P<0.01$, *** $P<0.001$, **** $P<0.0001$. **(B)** The graphs compile the amounts of the indicated proteins from *N*=11 independent Western blots. Statistical analysis was performed after logarithmic transformation due to a lognormal distribution of the values. Repeated measures one-way ANOVA with Tukey's multiple comparison was performed with * $P<0.05$, ** $P<0.01$, *** $P<0.001$.

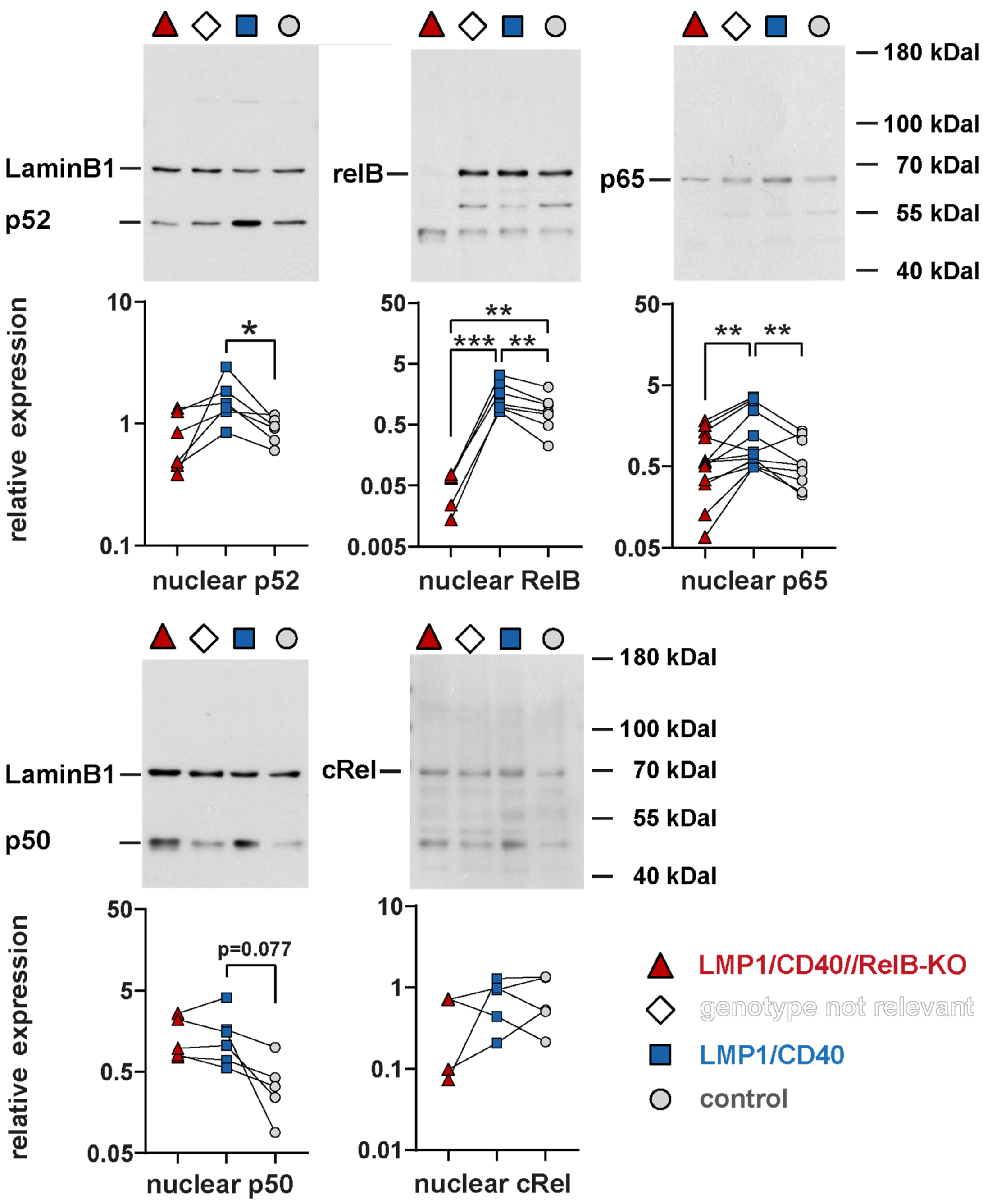

Supplementary Figure 6: Inactivation of RelB does not result in the hyperactivation of canonical NF-κB signaling in LMP1/CD40-expressing B cells. Western blots with nuclear extracts from splenic B cells from the indicated genotypes after staining with antibodies raised against RelB, p100/p52, p105/p50, p65 and c-Rel (top and bottom row one blot each). LaminB1 was used as loading control and for normalization of the quantified proteins. The graphs compile the amounts of the indicated proteins from $N \geq 4$ independent Western blots. Statistical analysis was performed after logarithmic transformation due to a lognormal distribution of the values. Repeated measures one-way ANOVA (or mixed-effects analysis, if single values were missing) with Tukey's multiple comparison was performed with * $P<0.05$, ** $P<0.01$, *** $P<0.001$.

the thin yellow line represents the outer borders of the

marginal sinus | complete follicle

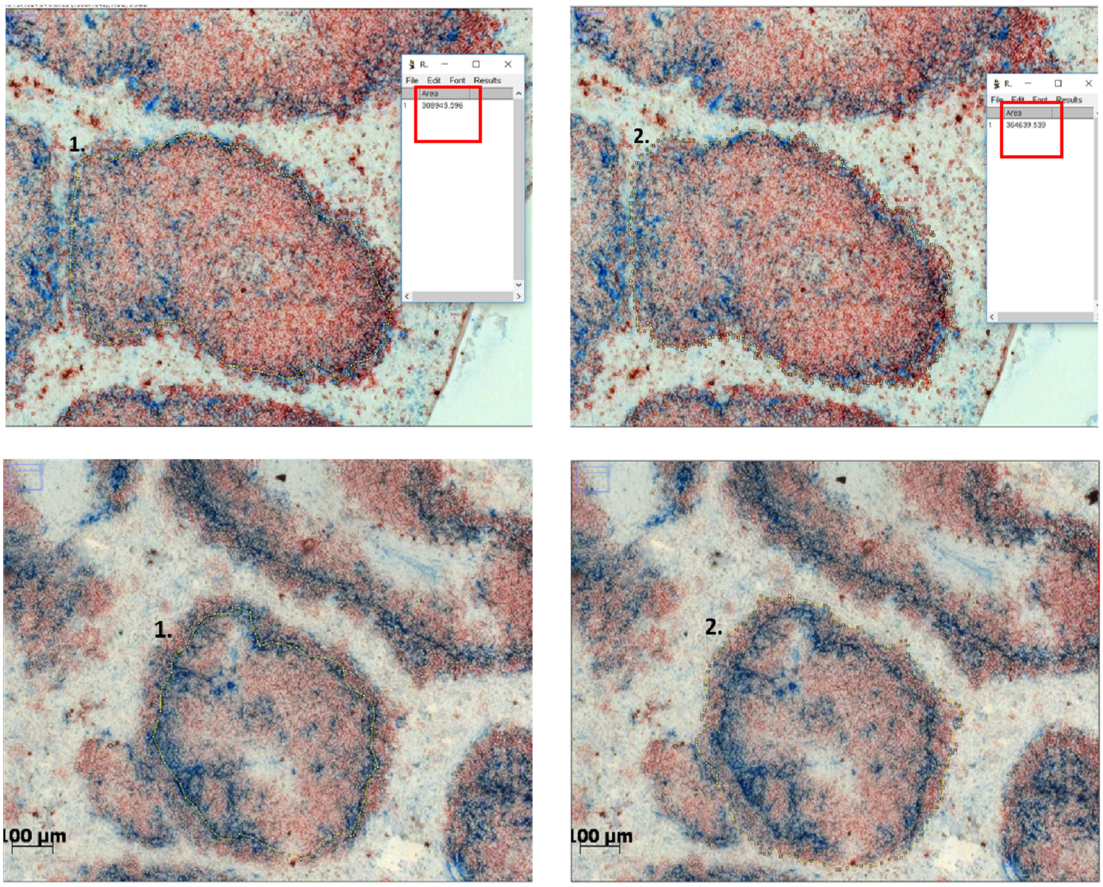

LMP1/CD40

LMP1/CD40 //RelB-KO

Supplementary Figure 7: RelB inactivation ablates the enhanced retention of LMP1/CD40-expressing MZB cells in the follicle. The figure shows an example for the determination of the MZ areas in spleens from LMP1/CD40 and LMP1/CD40//RelB-KO mice. B lymphocytes are stained in red with α-IgM. The outer borders of the marginal sinus (α-MOMA1, blue staining) and the complete follicle were manually set with ImageJ (see the yellow lines), to receive areas of the respective entities. The MZ areas result from the subtraction of both quantified areas, respectively.

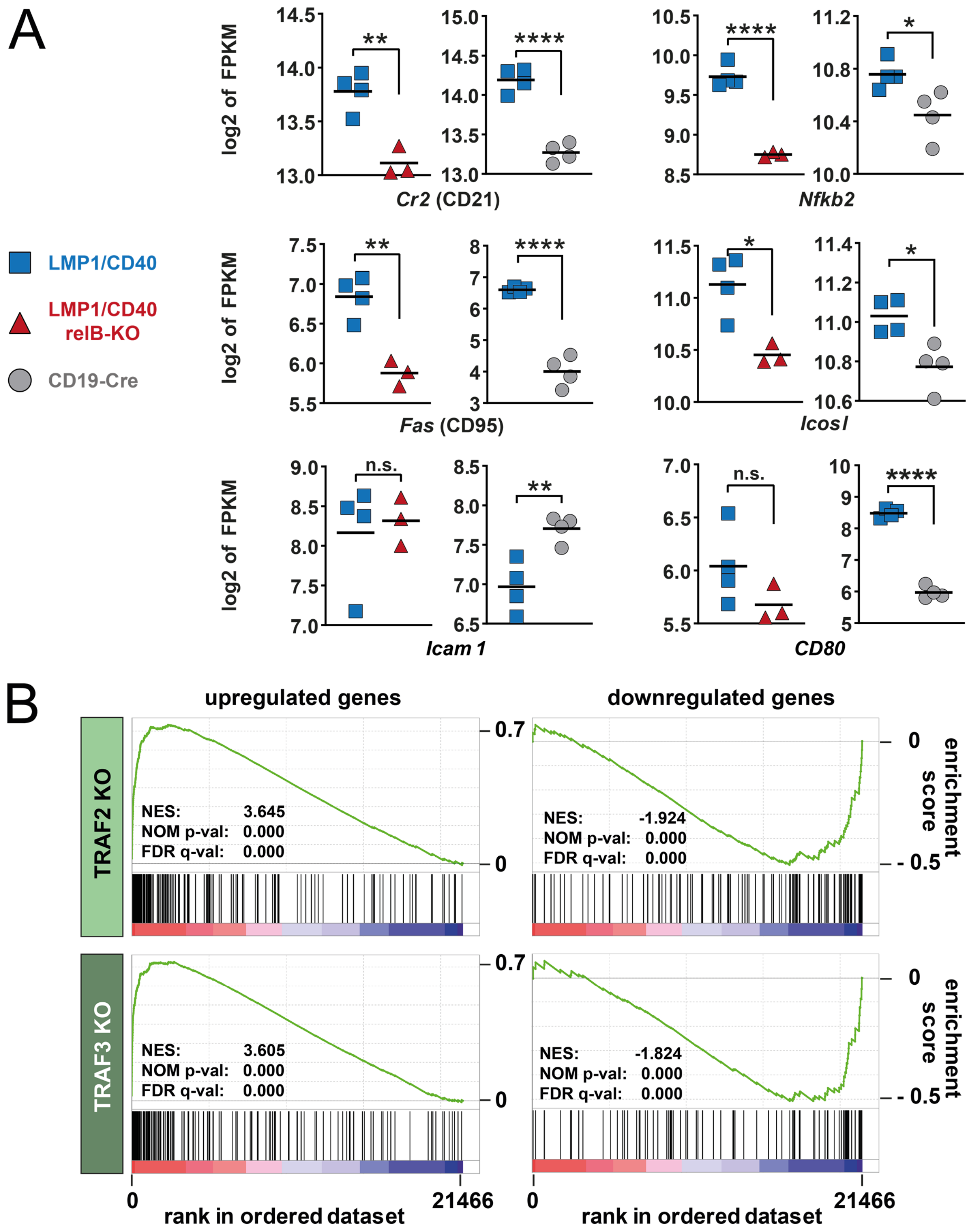

Supplementary Figure 8: Validation of the RNA-Seq analyses of splenic B cells from LMP1/CD40 vs LMP1/CD40//RelB-KO and LMP1/CD40 vs CD19-Cre mice. RNA-sequencing of B cells from *N*=4 LMP1/CD40 and *N*=3 LMP1/CD40//RelB-KO mice (comparison 1) and and from *N=4* LMP1/CD40 and *N=4* CD19-Cre mice (comparison 2) was performed. (**A**) For validation of the RNA-Seq analysis, expression levels of genes with known regulation at the protein level were examined. The graphs show the log2 of FPKM (fragments per kilobase of exon per million reads mapped) of the indicated genes and genotypes. Ordinary unpaired two-tailed t-tests were performed with * P<0.05, ** P<0.01, **** P<0.0001. **(B)** GSEA using TRAF2-KO and TRAF3-KO signature gene sets (up and down regulated, respectively) from B-lymphocytes derived from Gardam et al. (2008). The ordered dataset displays 21466 genes, ranked for fold changes from upregulated (left) to downregulated (right) in RelB proficient vs. RelB deficient LMP1/CD40 cells.

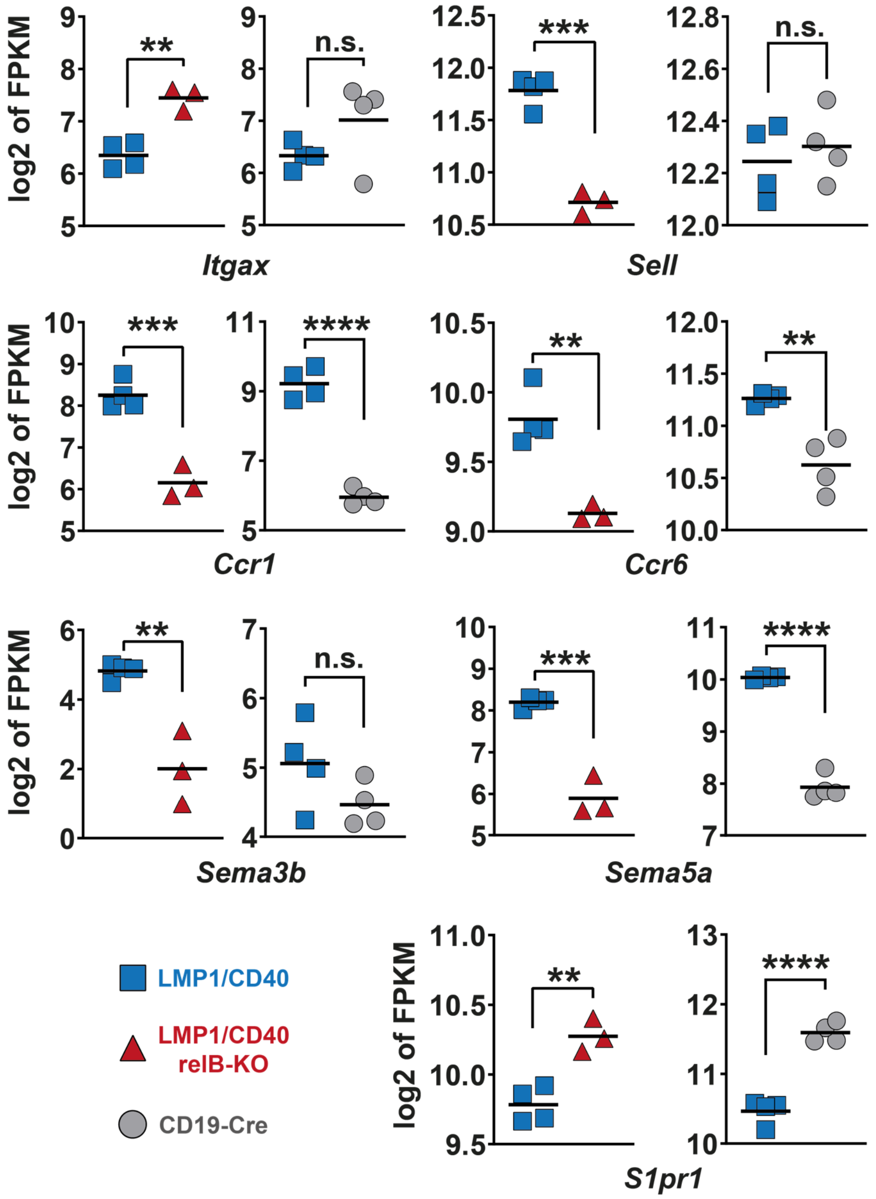

Supplementary Figure 9: The non-canonical NF-κB signaling pathway regulates genes involved in migration and cell adhesion. (A) RNA expression levels (log2 of FPKM) of the indicated genes in splenic B cells from LMP1/CD40 and LMP1/CD40//RelB-KO and from LMP1/CD40 and CD19-Cre mice. *N*=4 LMP1/CD40 and *N*=3 LMP1/CD40//RelB-KO (comparison 1) and from N=4 LMP1/CD40 and N=4 CD19-Cre mice (comparison 2). Ordinary unpaired two-tailed t-test was performed with ** P<0.01, *** P<0.001, **** P<0.0001.

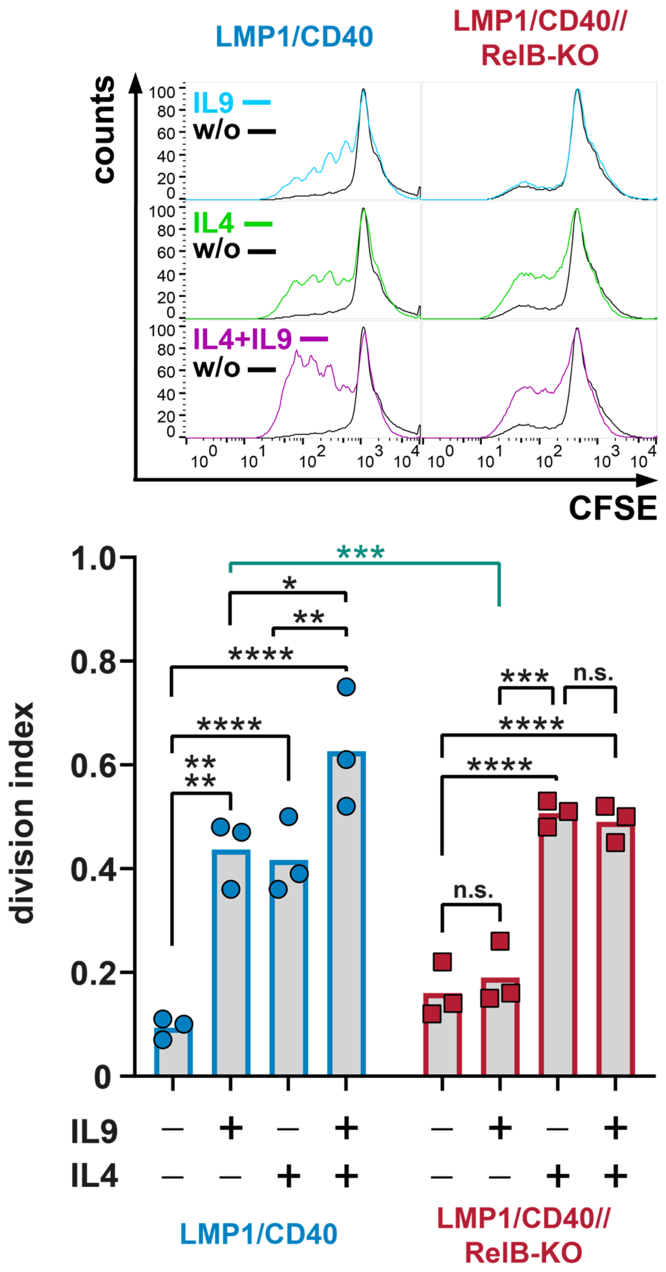

Supplementary Figure 10: RelB-proficient and –deficient B cells respond similarly to IL4 stimulation. The proliferation of splenic B cells isolated from mice with the indicated genotypes was analyzed after CSFE staining. Cells were kept either unstimulated or were stimulated with IL9 or IL4 or a combination of both for three days as indicated. Representative histogram overlays of the CFSE staining of stimulated and unstimulated B cells from the indicated genotypes and stimulations are shown. The histograms are pre-gated on live cells. The graphs compile the division rates in the individual samples from *N*=3 LMP1/CD40 and LMP1/CD40//RelB-KO mice. Statistics were calculated by a two-way ANOVA, Tukey´s multiple comparisons test with* P<0.05, ** P<0.01, *** P<0.001 and **** P<0.0001.

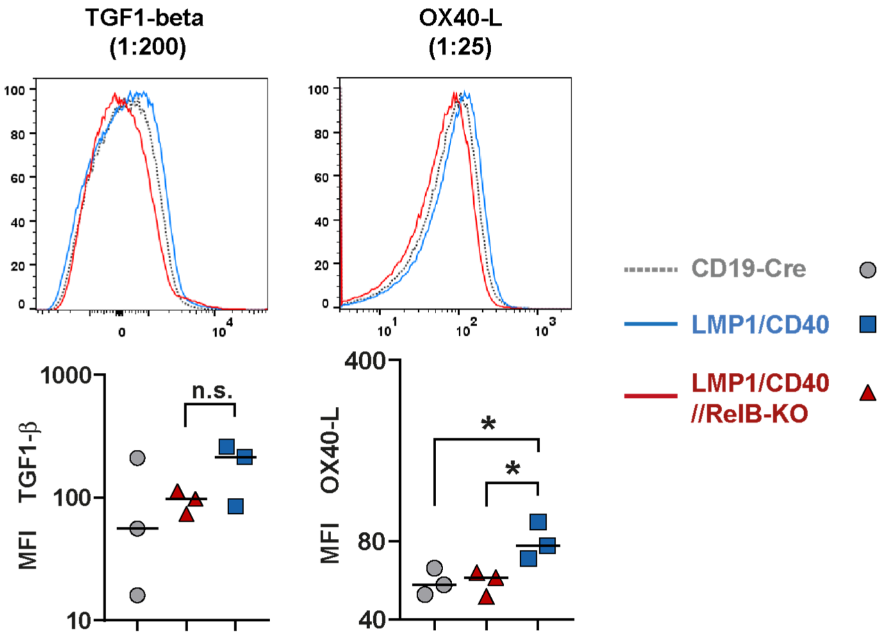

Supplementary Figure 11: OX40-L is higher expressed on B cells from LMP1/CD40, as compared to LMP1/CD40//RelB-KO and control mice. To elucidate why there are more IL-9 expressing $CD4^+$ T cells in LMP1/CD40 in comparison to LMP1/CD40//RelB-KO and control mice, we analysed the expression of ligands on the cell surface of splenic B cells known to be involved in the generation of Th9 cells. Representative histogram overlays are shown for TGF1-β and OX40-L. The histograms are pre-gated on live lymphocytes and $CD19^+$ cells. The graphs summarize the mean fluorescence intensities (MFI) of the indicated surface markers in the indicated genotypes from N=3 mice. Due to their log normal distribution, values were logarithmized before calculating the statistics with ordinary one-way ANOVAs with Tukey's multiple comparison. * $P<0.05$, Symbols represent values from individual mice, and the bars indicate means. The experiments were done with animals with an age of 7-10 months, since younger mice were not available at the time of analysis (experiment was done during revision).

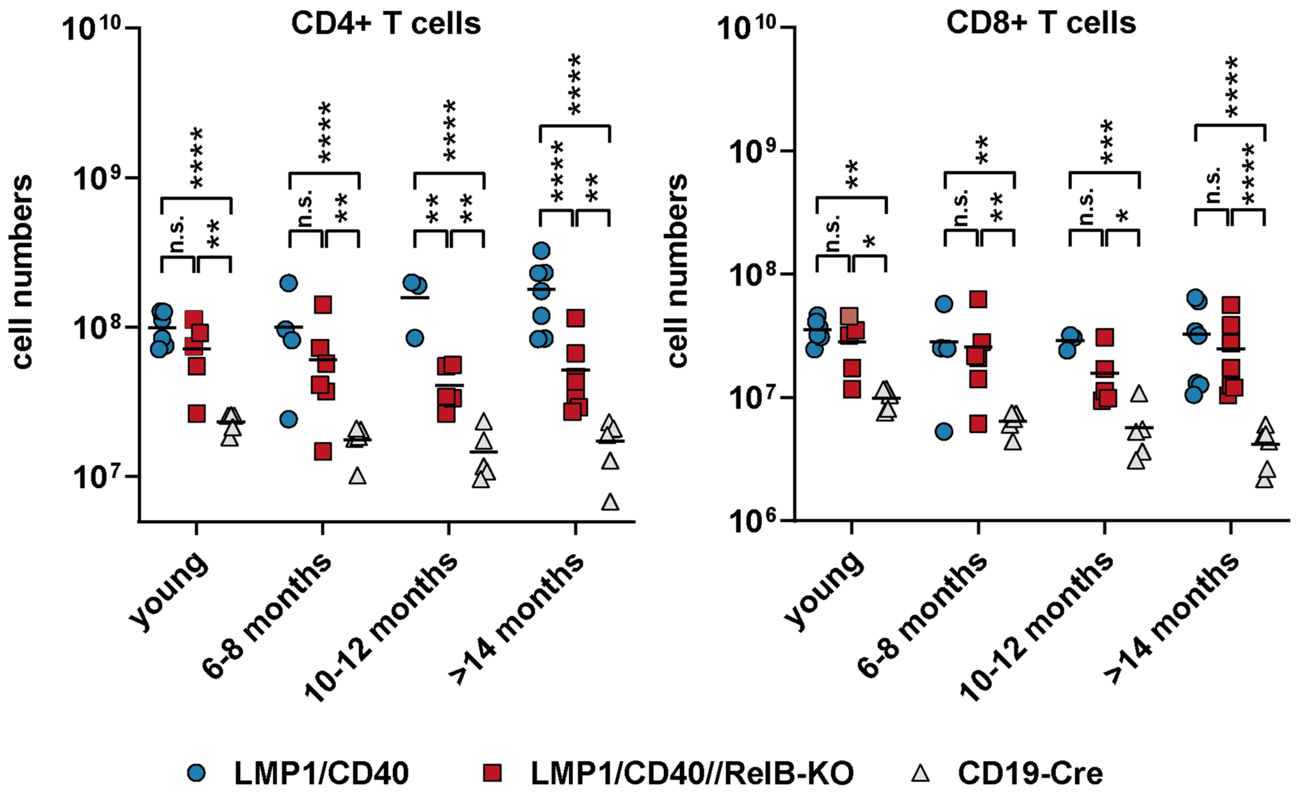

Supplementary Figure 12: Total numbers of CD4$^+$ T cells increase more in LMP1/CD40 in comparison to LMP1/CD40//RelB-KO mice during aging. T cell numbers were determined in the spleen of LMP1/CD40 (blue) LMP1/CD40//RelB-KO (red) and control (grey) mice at different ages. Due to their lognormal distribution cell numbers were logarithmized before calculating the statistics. Statistical analysis was performed with two-way ANOVA with Tukey's multiple comparison. Dots represent values from individual mice (*N≥3*), and the lines and numbers indicate the means. * P<0.05, ** P<0.01, *** P<0.001, **** P<0.0001.

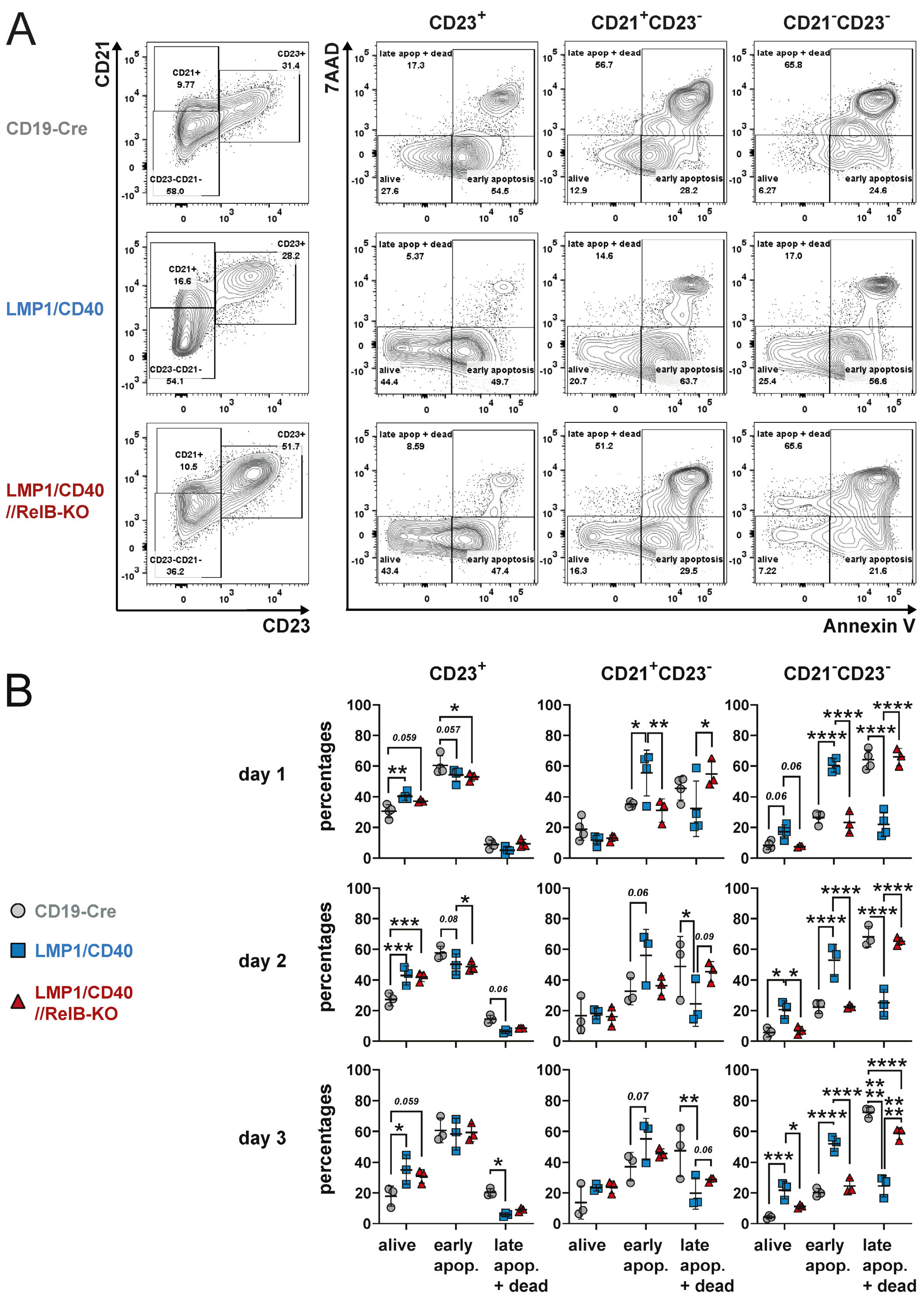

Supplementary Figure 13: The CD21^{-}CD23^{-} population is more highly apoptotic in LMP1/CD40//RelB-KO in comparison to LMP1/CD40 mice. B lymphocytes were enriched from the total splenic cell population from 7-10 months old mice with the pan-B cell kit. The isolated B cells were taken in culture for 72 h. At days 1, 2 and 3 cells were stained for CD23 and CD21 in combination with apoptosis markers Annexin V and 7AAD. **(A)** Exemplary FACS plots with B cells stained at day 2. FACS plots are pre-gated on CD19^{+} B cells and subsequently separated into CD23^{+}CD21^{+}, CD23^{-}CD21^{+} and CD23^{-}CD21^{-} B cells. Viable cells (Annexin V^{-}7AAD^{-}), early apoptotic cells (Annexin V^{+}7AAD^{-}), as well as late apoptotic and dead cells (Annexin V^{+}7AAD^{+}) were gated in each of the 3 populations. FACS analyses are representative for *N=4* control mice, *N=4* LMP1/CD40 mice and *N=3* LMP1/CD40//RelB-KO mice. **(B)** The graphs compile the percentages of alive, early apoptotic and late apoptotic/dead cells on day 1, 2 and day 3. Statistical analysis was performed with an ordinary two-way ANOVA with Tukey's multiple comparison. * P<0.05, ** P<0.01, *** P<0.001, **** P<0.0001

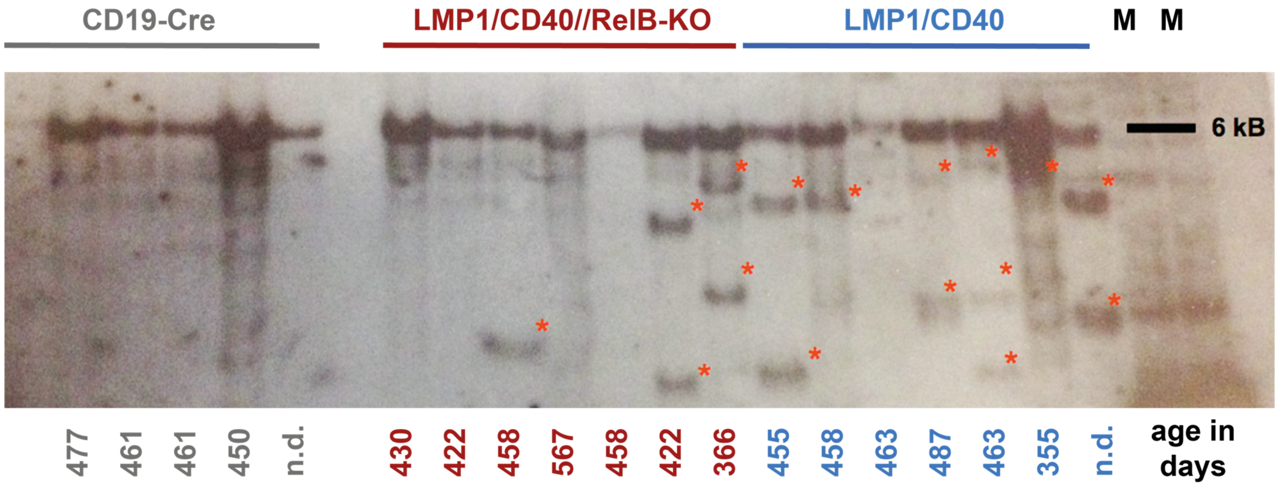

Supplementary Figure 14: Lymphoma development is reduced in RelB-deficient LMP1/CD40 mice. Representative Southern blot analysis with EcoRI digested DNA, which was prepared from splenic cell suspensions. The blot was hybridized with a radioactive labelled probe from the J_H1-4 immunoglobulin region. The heavy chain in the germline configuration is detected as a 6kb fragment. Additional bands marked with red stars indicate monoclonal B cell populations. Below the blot the respective age of the mice is indicated.

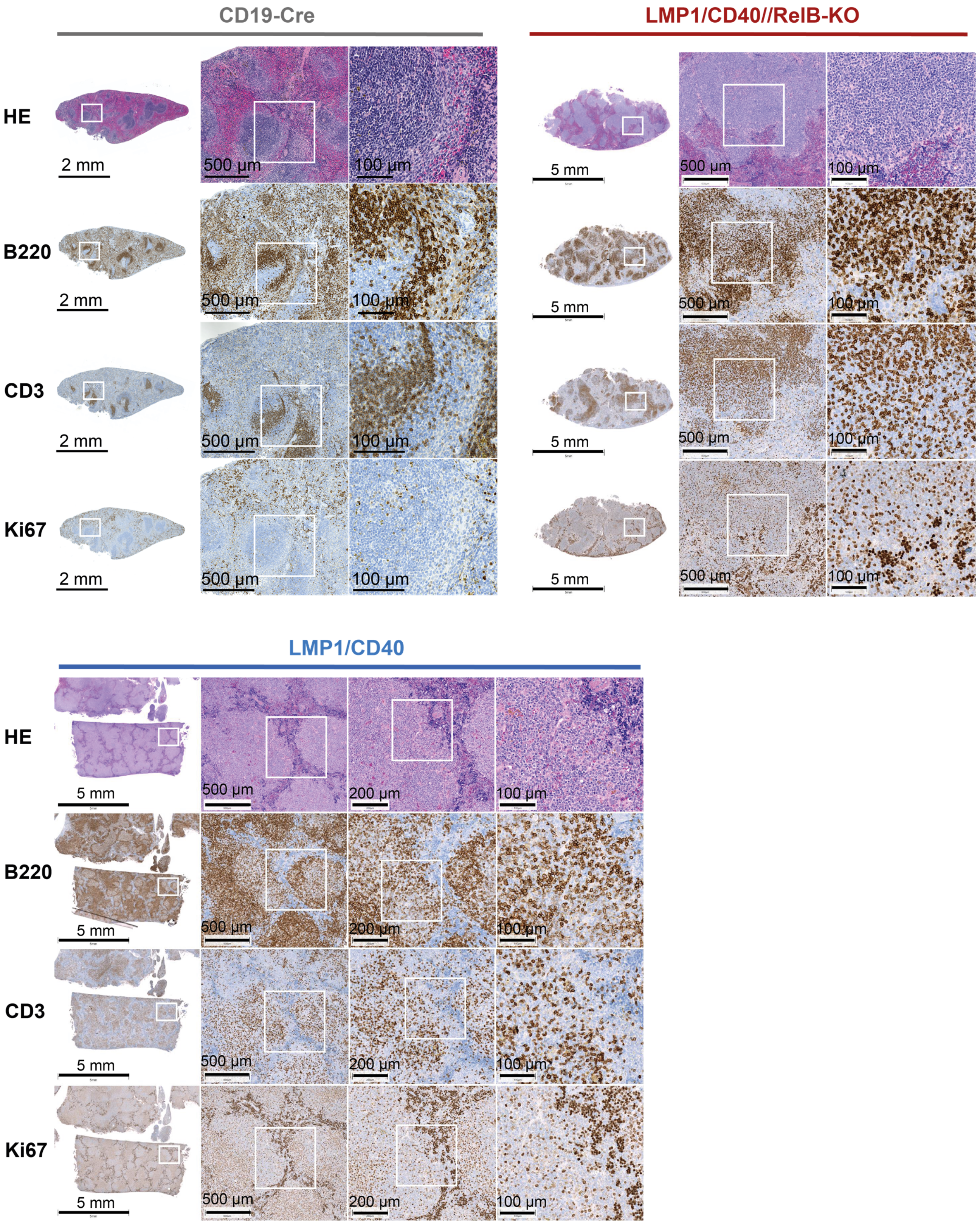

Supplementary Figure 15: The splenic architecture is disrupted in diseased aged LMP1/CD40 and LMP1/CD40//RelB-KO mice. Exemplary immunohistochemistry of diseased LMP1/CD40 and LMP1/CD40//RelB-KO mice. Splenic paraffin sections were stained with HE to examine the splenic architecture. B cells and T cells were detected after a B220-staining and CD3-staining respectively. To detect proliferating cells, tissue sections were stained with an anti-Ki-67 antibody. Control mice were analysed at the end of our observation time and were still healthy. Age of mice: LMP1/CD40 405 days, LMP1/CD40//RelB-KO mice 365 days, CD19-Cre 513 days.

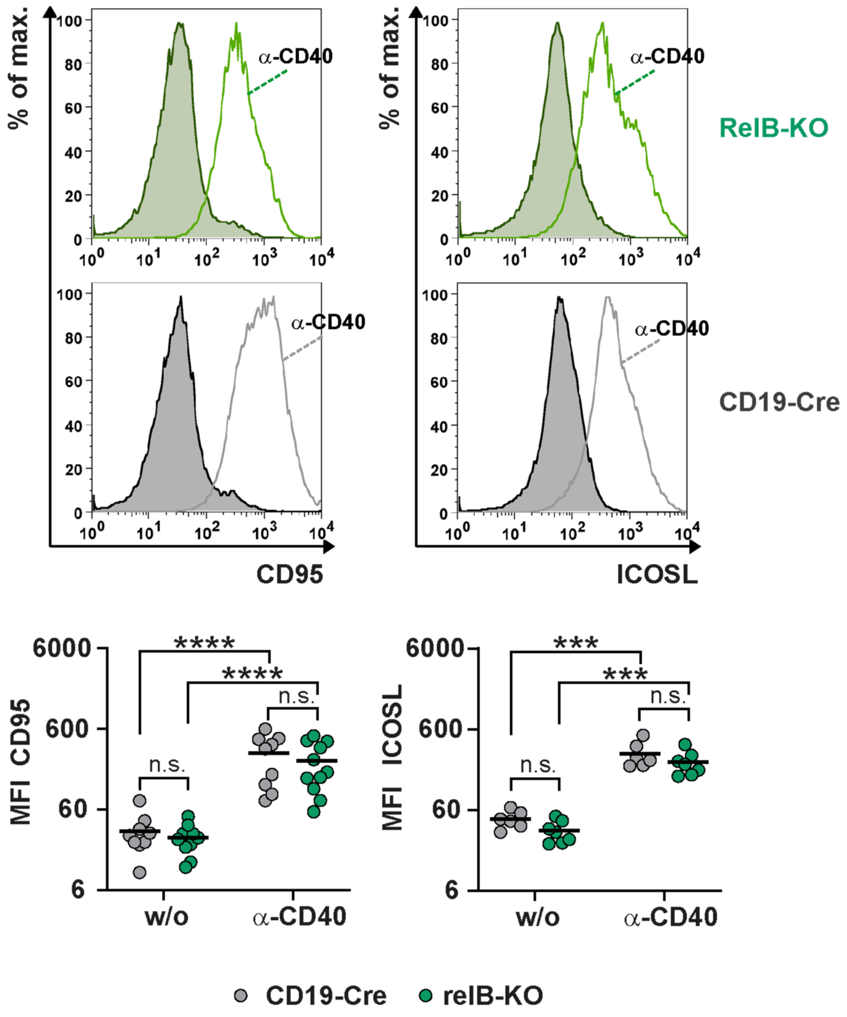

Supplementary Figure 16: RelB-deficient B cells can be activated by CD40-stimulation in vitro. Splenic MACS-purified B cells from control and RelB-KO mice were stimulated with an agonistic CD40 antibody for three days before they were analyzed for their CD95 and ICOSL expression. The histograms show an overlay of stimulated (d3) and unstimulated B cells (d0). FACS histograms are pre-gated on living cells. The graph compiles the MFIs from N≥6 independent experiments. The values were logarithmized before calculating the statistics by mixed-effect analysis with Tukey´s multiple comparison test.

Supplementary Table 1: Primers

PCR	Oligonucleotide	Sequence (5'-3')
CD19	Cre7	tca gct aca cca gag acg g
	CD19c	aac cag tca aca ccc ttc c
	CD19d	cca gac tag ata cag acc ag
LMP1/CD40 (CD40)	CD40 PCR3	ctg aga tgc gac tct ctt tgc cat
	LMP1ex1fw1	agg agc cct cct tgt cct cta
rosa wt locus	Rosa 60	tac tcc gag gcg gat cac aag c
	Rosa 62	ctc tcc caa agt cgc tct g
RelB	RelB-GT 1	agg ttg atg gta act ttg
	SeqTVRelB1	tcc aaa aaa acc aaa cca
	SeqTVRelB8	gtt ttc cct gct tgg ttc

Target	Primer sequence (5'-3')	Roche probe number
YWHAZ	cgc taa taa tgc agt tac tga gag aga	2
	ttg gaa ggc cgg tta att tt	
RNA POL2	aat ccg cat cat gaa cag tg	69
	tca tca tcc att tta tcc acc a	
LILRB4/CD85k	tggagtcctggtgtcattcc	69
	tgtgtgttcttcacagaagcatt	
IL9R	ggacagttggcagtaagtcacc	20
	ccactctctccaaggtccaa	
ACKR3/CXCR7	gcaagagatggccaagagac	68
	cttgaggagagcgaccaagt	
CNR1	gggcaaatttccttgtagca	79
	ggctcaacgtgactgagaaa	

Supplementary Table 2: Antibodies for FACS

Epitope	Clone	Company
AA4.1	AA4.1	eBioscience
B220	RA3-6B2	BD Biosciences
CD4	RM4-5	BD Biosciences
CD5	53-7.3	BD Biosciences
CD19	1D3	BD Biosciences
CD21	7G6	BD Biosciences
CD23	B3B4	BD Biosciences
CD43	S7	BD Biosciences
CD80	16-10A1	BD Biosciences
CD95	Jo2	BD Biosciences
ICAM	3E2	BD Biosciences
ICOS-L	HK5.3	eBioscience
IgM	R6-60.2	BD Biosciences
IgD	R26-46	BD Biosciences
IL9	RM9A6	eBioscience
IL9-R	N.A.	Kindly gifted from Kitamura Lab
Isotype	eBRG1	BD Biosciences
LiLRB4/CD85k	H1.1	BD Biosciences
Lymphotoxin ß-R	eBio3C8	eBioscience
OX40-L	OX89	Invitrogen
TGF-β1 (LAP)	TW7-16B4	BioLegend

Supplementary Table 3: Antibodies for Western blots and histology

Epitope	Conjugate	Clone or Cat.Nr (#)	Company
RelB	-	#4922S	Cell Signaling
p100/p52	-	#4882S	Cell Signaling
P105/p50		#sc-2882	Santa Cruz
IκB-α		#sc-371	Santa Cruz
pIκB-α		#9246S	Cell signaling
p65		#4764	Cell signaling
c-Rel		#sc-6955	Santa Cruz
GAPDH	-	6C5	Merck millipore
Tubulin	-	#2148S	Cell Signaling
Lamin B1		#sc-6217	Santa Cruz
Lamin B2	-	E1S1Q	Cell Signaling
Anti-rabbit	HRP	7074S	Cell Signaling
Anti-mouse	HRP	7076S	Cell Signaling
Anti-mouse IgM	peroxidase		Sigma-Aldrich
Rat-anti-mouse CD3			homemade
Rat-anti-mouse Moma1		T2011	BMA Biomedicals AG
Mouse anti Rat IgG2	Biotin		Jackson Laboratories
Anti-CD45R (paraffin		#550286	BD Pharmigen
Rabbit anti-CD3 (paraffin)		#ab1669	Abcam
Rabbit Anit-Ki67 (paraffin)		#ab15580	Abcam
